# *Aedes albopictus* diversity and relationships in south-western Europe and Brazil by rDNA/mtDNA and phenotypic analyses: ITS-2, a useful marker for spread studies

**DOI:** 10.1186/s13071-021-04829-9

**Published:** 2021-06-26

**Authors:** Patricio Artigas, Marta Reguera-Gomez, María Adela Valero, David Osca, Raquel da Silva Pacheco, María Goreti Rosa-Freitas, Teresa Fernandes Silva-do-Nascimento, Claudia Paredes-Esquivel, Javier Lucientes, Santiago Mas-Coma, María Dolores Bargues

**Affiliations:** 1 Departamento de Parasitología, Facultad de Farmacia, Universidad de Valencia, Av. Vicent Andrés Estellés s/n, 46100 Burjassot, Valencia Spain; 2 Laboratõrio de Pesquisa Clínica e Vigilância em Leishmanioses, Instituto Nacional de Infectologia Evandro Chagas, INI, FIOCRUZ, Rio de Janeiro, Brazil; 3 Laboratório de Mosquitos Transmissores de Hematozoários, Instituto Oswaldo Cruz, Fiocruz, Rio de Janeiro, Brazil; 4 Grupo de Zoología Aplicada y de La Conservación, Departamento de Biología, Universidad de las Islas Baleares, Palma de Mallorca, Spain; 5 Instituto de Investigación Agroalimentario de Aragón IA2, Facultad de Veterinaria, Universidad de Zaragoza, Zaragoza, Spain

**Keywords:** *Aedes albopictus*, Molecular haplotyping, rDNA 5.8S-ITS-2, mtDNA *cox*1, Cloning, Sequencing, Wing geometric morphometry, Disease vector, South-western Europe, Brazil

## Abstract

**Background:**

*Aedes albopictus* is a very invasive mosquito, which has recently colonized tropical and temperate regions worldwide. Of concern is its role in the spread of emerging or re-emerging mosquito-borne diseases. *Ae. albopictus* from south-western Europe and Brazil were studied to infer genetic and phenetic diversity at intra-individual, intra-population and inter-population levels, and to analyse its spread.

**Methods:**

Genotyping was made by rDNA 5.8S-ITS-2 and mtDNA *cox*1 sequencing to assess haplotype and nucleotide diversity, genetic distances and phylogenetic networks. Male and female phenotyping included combined landmark-and outlined-based geometric morphometrics of wing size and shape.

**Results:**

Specimens from seven populations from Spain, France and Brazil provided 12 *cox*1 and 162 5.8S-ITS-2 haplotypes, with great genetic variability difference between both markers (0.9% vs 31.2%). Five *cox*1 haplotypes were shared with other countries, mainly Italy, USA and China, but none was shared between Europe and Brazil. The 5.8S-ITS-2 showed 2–7 intra-individual (mean 4.7) and 16–34 intra-/inter-population haplotypes (24.7), including haplotypes shared between Spain, France and Brazil. A 4.3% of ITS-2 haplotypes were shared, mainly with Italy, USA and Thailand, evidencing worldwide spread and introductions from areas where recent outbreaks of *Ae. albopictus*-transmitted pathogens occurred. Wing size showed sex differences. Wing shape distinguished between Brazilian and European specimens. Both genetic and morphometric markers showed differences between insular Spain and continental Spain, France and Brazil.

**Conclusions:**

ITS-2 proves to be a useful marker to assess *Ae. albopictus* spread, providing pronouncedly more information than *cox*1, including intra-individual, intra-population and inter-population levels, furnishing a complete overview of the evolutionary exchanges followed by this mosquito. Wing morphometry proves to be a useful phenotyping marker, allowing to distinguish different populations at the level of both male and female specimens. Results indicate the need for periodic surveillance monitorings to verify that no *Ae. albopictus* with high virus transmission capacity is introduced into Europe.

**Graphic Abstract:**

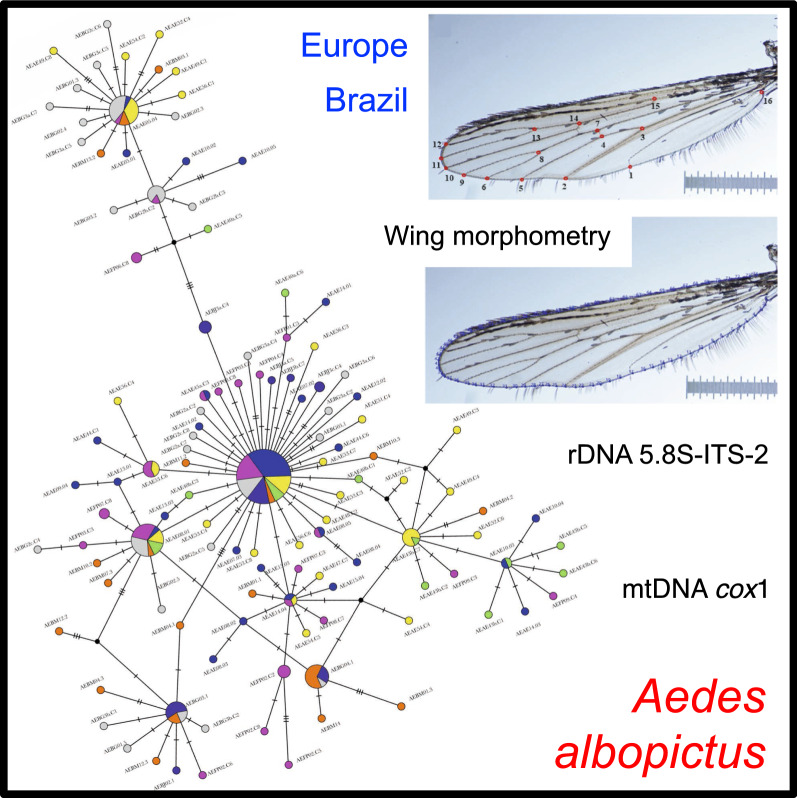

**Supplementary Information:**

The online version contains supplementary material available at 10.1186/s13071-021-04829-9.

## Background

One of the most important current health alerts is the risk of spread of vector-borne diseases due to the effects of climate change and global change. Vector-borne diseases are human illnesses caused by parasites, viruses and bacteria that are transmitted by vectors and cause every year more than 700,000 deaths [1]. Over the last decade, a number of these diseases have emerged or re-emerged in southern Europe, including 5 viral, 1 bacterial, 3 protozoan and 2 helminth infections [2]. Of particular concern is the emergence or re-emergence of mosquito-borne diseases, affecting both humans an animals mainly in tropical and subtropical countries, even leading to worrying increases of incidence in temperate regions [3, 4].

The Asian tiger mosquito *Aedes *(*Stegomyia*)* albopictus* (Skuse, 1894) is one of the most invasive species which has successfully colonized most tropical and temperate regions around the world [5]. This species is of importance in public health, not only for its role in the transmission of various pathogens (arboviruses and parasites) to humans, but also for its considerable amount of nuisance biting in areas where it is well-stablished, reducing the quality of life of inhabitants [6], especially those who develop their activities outdoors.

The geographic distribution of *Ae. albopictus* was restricted to the islands of the Indian Ocean and the islands in the Pacific Ocean until the 1970s, when an expansion of its geographical distribution rapidly occurred throughout all continents, except the Antarctica [7]. This phenomenon was likely primarily driven by the transportations of drought-resistant eggs via international trade [8]. Its adaptation to colder climates may result in disease transmission in new areas.

In Europe, the first record of this species was in 1979 in Albania [9]. Since its finding in Italy in 1990, *Ae. albopictus* is known to be spreading in several European countries such as the Balkan countries, Greece, France, and Spain, where this species established along the Mediterranean coast [10–12]. At present, *Ae. albopictus* has been recorded in 27 European countries and has become established in 19 of them [13–16].

Since the arrival of the tiger mosquito to Spain in 2004 [17], the number of invaded territories has increased despite vector control surveillance efforts. The Mediterranean coastal areas are the most affected territories in the country [18]. In the Balearic Islands, it was first detected in Mallorca in 2012 [19], with a rapid colonization by the species [20, 21], and subsequent reports in Ibiza (2014) and Minorca (2016) [11, 22]. Touristic resorts proved to be strong predictors of the presence of *Ae. albopictus* in the Balearic Islands [20]. Even when islands are particularly vulnerable to the incursion of invasive species, there is not sufficient information on the population genetics of *Aedes* mosquitoes in these ecosystems. In 2017, *Ae. albopictus* was reported for the first time in northern Portugal [23] and recently in Extremadura, western Spain [24]. Although its worldwide colonization has been rapid, and most probably due to passive transportation, in Spain there is direct evidence about *Ae. albopictus* having been dispersed by vehicles, with Barcelona as the largest source of inter-province tiger mosquito transfers [18]. Similar mosquito arrivals have been reported in UK in vehicles from continental Europe through the ferry ports and Eurotunnel [13]. Human transport networks have been highlighted for their role in the dispersal and establishment of *Ae. albopictus* in Europe [25].

The distribution of this invader in South America follows a similar pattern of rapid expansion. Detected in Brazil during the 1990s, it subsequently spread to Venezuela, Colombia, Argentina, Bolivia, Paraguay, Uruguay and recently Ecuador [26]. *Ae. albopictus* was first recorded in Brazil in 1986, in the states of Rio de Janeiro and Minas Gerais [27] and now is currently present in 26 of 27 Brazilian federative units [28].

Molecular markers generate a large amount of information about genetic diversity and phylogenetic relationships of the organisms. In insects, DNA markers prove to be useful for this endeavour in general, although disadvantages and limitations of each marker should be taken into account [29, 30]. In *Ae. albopictus*, several genetic markers (mtDNA, microsatellites, allozymes) have been used to study population genetics. Everything seems to indicate the existence of a global chaotic pattern of *Ae. albopictus* distribution, with several introductions and admixture between unrelated genomes [31], and a genetic variation within the populations that largely contributes to the total genetic diversity [32]. The few studies that included samples from Europe showed several genotypes, which were compatible with multiple introduction events into Europe [31, 33–35].

Internal transcribed spacers (ITSs) of the nuclear ribosomal DNA (rDNA) have proved to be useful for the classification of species, subspecies, hybrids, and populations of different groups of insects and for inferring their phylogenetic relationships [29]. The ITS-2 spacer has a higher rate of mutation than most of the nuclear rDNA operon. It accumulates mutations within isolated populations relatively quickly and has been extensively used for species identification [29, 36, 37]. Indeed, ITSs are not subject to the same functional constraints as the rRNA genes and are therefore subject to higher evolutionary rates leading to greater variability in both nucleotide sequence and length. Despite not being a gene, the ITS-2 develops a key function in rRNA maturation, so that even minor modifications to this spacer can inhibit or prevent the formation of mature rRNA products [29, 38].

Opposite to the clonal maternal inheritance of mtDNA genes, ITS-2, together with the whole nuclear ribosomal DNA operon, follows Mendelian inheritance and is therefore of interest in biparental organisms such as mosquitoes. Moreover, it presents the peculiarity of following a concerted evolution. With sufficient time, this mechanism effectively homogenizes the many copies of nuclear rDNA among both homologous and non-homologous chromosomes containing rDNA clusters within the genome. This gives rise to a uniformity inside all individuals of a population and becomes extremely useful [29, 38]. Although exceptions have been described in given organism cases, the concerted evolution usually applies, as in the case of *Aedes* mosquitoes.

Because of concerted evolution, when two different ITS-2 sequences are found in a specimen collected in nature, it indicates that it is a hybrid, either (i) the direct result of a crossbreeding between parental specimens belonging to two different populations (differing in haplotype profile), subspecies (as in geographical border areas), or two close species (as in cryptic species living in sympatry), or (ii) a descendant of a viable hybrid lineage originated so recently that concerted evolution has not yet had the time needed for homogenisation [29]. This kind of intraindividual heterogeneity can be elucidated by obtaining the original parent sequences mixed inside an individual by means of cloning. Such cases can be explained by separated origin (different geographical areas and/or different ecological habitats) of the evolutionary units which undergo separated divergences and are subsequently put in local sympatry after geographical spreading of one or the two entities giving rise to overlapping distribution (mainly due to human intervention) in which hybridisation takes place when the evolutionary units have not yet reached complete reproductive or genetic isolation [29].

In *Ae. albopictus*, ITS-2 has been also used as indicator of discontinuity between populations and thus used to address the presence of intraspecific differentiation (genetic barriers) among groups of *Ae. albopictus* populations from different ancestral and recently colonized geographic areas [39].

Different mtDNA regions or some complete mitogenomes have been used to study the genetic structure and analyse the phylogeography of *Ae. albopictus* [33, 40–42]. Within mtDNA, the *cytochrome oxidase I* (*cox*1) gene is the most widely used and is the gold standard for mosquito species identification [43]. However, different authors have used fragments from different regions of the *cox*1, leading to difficulties in comparisons of different populations [32].

In the present work, we use rDNA/mtDNA genotyping and wing morphometry phenotyping to analyse populations of *Ae. albopictus* from temperate regions of Western Mediterranean Europe and tropical regions of Brazil to assess their relationships. Research focused on haplotype and nucleotide diversity as well as genetic distances and phylogenetic networks combined with landmark- and outlined-based geometric morphometrics of wings. The aim was to infer genetic and phenetic diversity at intra-population and inter-population levels, as well as the genetic diversity at intra-individual level, of European and Brazilian populations of this vector to contribute to the understanding of vector spread and disease transmission risks.

## Materials and methods

### Mosquito collections

Samples of *Ae. albopictus* were collected between 2014 and 2018 in three countries, representing different localities in south-western Europe and Brazil, and analysed genetically and morphologically (Table 1). The specimens from south-western Europe were collected in several localities of the Mediterranean Basin of Spain (Valencia and Barcelona provinces) and on the island of Mallorca, Balearic Archipelago, as well as in Perpignan, region of Pyrénées-Orientales, in the Southern part of France. The mosquitoes from Brazil were collected in three regions: Manaus, Jurujuba and Goiania (Amazonas State, Rio de Janeiro State, Goias State, respectively) (Table 1).

**Table 1:** Mosquito samples and their geographical locality of origin analysed for genotyping and phenotyping

	Brazil			Spain			France	Total
	Goiânia, Goiás State	Jurujuba, Niterói, Río de Janeiro State	Manaus, Amazonas State	Barcelona, Catalunya Province	Valencia, Valencia Province	Mallorca, Balearic Archipielago	Perpignan, Pyrénées-Orientales	
Geographic coordinates	16°40′44″S 49°15′14″W	22°55′57.36"S 43°6′54.53"W	3°06′00″S 60°01′00″W	41°23′11.32"N 2°10′22.94"W	39°28′11.1"N 0°22′38.3"W	39°37′3.25"N 2°43′1.52"E	42°40′51.66"N 2°54′3.17"E	
Date	2016	2016	2016	2017^a^	2016–2017	2014–2017	2018	
Climate	Tropical	Tropical	Tropical	Temperate	Temperate	Temperate	Temperate	
Genotyping:	11	11	14	14	26	25	10	111
Males	7	8	10	7	14	15	1	62
Females	4	3	4	7	10	10	9	47
L/P					2			2
Phenotyping:	10	11	13	14	23	21	11	103
Males	6	8	9	7	15	12	2	59
Females	4	3	4	7	8	9	9	44
Both analyses:	10	11	13	14	22	21	10	101
Males	6	8	9	7	14	12	1	57
Females	4	3	4	7	8	9	9	44

^a^From Laboratory colony of San Cugat del Vallés (Baix Llobregat, Barcelona, Spain)

L/P, larval and pupal stages of *Ae. albopictus*

Adult mosquitoes from Spain were collected using oviposition traps [20, 44] (Mallorca), or directly collected as larvae or pupae, in natural reservoirs of water, and reared to adults under natural conditions (Valencia). Specimens from Barcelona were descendants of specimens collected in 2009, in the locality were *Ae. albopictus* was recorded for the first time in Catalonia, North-eastern Spain (Sant Cugat del Vallés, Baix Llobregat, Bacelona), and reared in laboratory colony under controlled conditions of 25 ± 1 °C, 75 ± 5% relative humidity and a 12 h/12 h light/darkness photoperiod [45]. Adult mosquitoes from France, and several from Spain (Valencia), were caught directly inside human dwellings. The populations of *Ae. albopictus* from Brazilian localities were obtained by spawning of females collected in oviposition traps in the aforementioned localities. The traps were brought to the laboratory under biosafety conditions required by current legislation. All material was sorted and the straws containing the eggs were transferred to vats containing water for hatching of the larvae. Emerging adults were identified and separated into breeding cages. The specimens were raised in the Laboratório de Mosquitos Transmissores de Hematozoários / IOC-Fiocruz, under controlled temperature and humidity.

The specific classification of specimens was made by using a dichotomic key [46] and genetically confirmed by sequencing of rDNA and mtDNA markers, as described below.

### Molecular analyses

#### DNA sequencing

A total of 111 *Ae. albopictus* representing seven populations from three countries were included in molecular analyses (Table 1). DNA was extracted from 2 to 3 legs of each mosquito, preserved in 70% ethanol at room temperature after collection, using InstaGene™ Matrix kit (Bio-Rad Laboratories® CA, USA) following the manufacturer's instructions. Two molecular markers were used for mosquito haplotyping: the internal transcribed spacer 2 (ITS-2) of the rDNA and the cytochrome C oxidase subunit 1 (*cox*1) of the mtDNA.

Each one of the DNA markers were PCR amplified independently for each specimen and each PCR product was sequenced for a bona-fide haplotype characterization. The complete rDNA ITS-2 was amplified using primers and PCR conditions previously described [47–50]. The almost complete *cox*1 gene was amplified with 2 sets of primers [51]. Each 25 μl reaction included 3–5 μl of DNA, 0.5 mM MgCl_2_, 0.2 mM dNTPs, 10 pmol of each primer and 0.5 U of DNA Polymerase (Biotools, Madrid, Spain). Amplification procedures and thermal cycler conditions for each one of the DNA markers were carried out in a Mastercycle ep*gradient* (Eppendorf, Hamburg, Germany), as previously described [48, 51].

When sequencing the *Ae. albopictus* specimens, the decision to go for ITS-2 cloning was taken already from the beginning, when double peaks were observed in many positions in the electropherograms of the ITS-2 sequences obtained by direct specimen sequencing. No one such situation of double peaks was observed in the *cox*1 sequences obtained. In the case of ITS-2, the PCR product obtained was cloned with pGEM-T Easy Vector System I (Promega, Madison, WI) and introduced in *Escherichia coli* DH5α competent cells. After the growth of colonies, standard PCR of 2–8 different colonies per sample was performed and individually sequenced. This cloning allowed for the obtaining of a DNA sequence including half the length of the 5.8S gene followed by the complete ITS-2 sequence, in its turn followed by ~ 20 nucleotides of the initial 5' region of the 28S gene.

PCR products were purified using the Ultra Clean™ PCR Clean-up DNA Purification System (MoBio, Solana Beach, CA, USA) according to the manufacturer's protocol and resuspended in 50 μl of 10 mM TE buffer (pH 7.6). The final DNA concentration (in μg/ml) and the absorbance at 260/280 nm were determined in a Eppendorf BioPhotometer  (Eppendorf, Hamburg, Germany).

Sequencing was performed on both strands by the dideoxy chain termination method carried out with the Taq dye-terminator chemistry kit on an Applied Biosystems 3730xl DNA Analyser (Applied Biosystems, Foster City, CA, USA) using the same amplification PCR primers.

#### Sequence analysis

Sequences were edited and assembled using Sequencher v5.4.6. (Gene Codes Co. MI, USA) and aligned using CLUSTALW2 [52], using default settings. The quality of sequencing products was assessed by visualization and checking of each chromatogram obtained for both forward and reverse primers of each marker (ITS-2 and *cox*1). Minor corrections for a better fit of nucleotide or indel (insertion/deletion) correspondences were made in the cases of the ITS-2 spacer. Aligned sequences were collapsed to haplotypes, counting gaps as differences, using the ALTER web server [53]. Homologies were performed using the BLASTN programme from the National Center for Biotechnology Information website (http://www.ncbi.nlm.nih.gov/BLAST). Genetic distances were measured using parameters provided by PAUP v.4.0a (build166) [54]. Molecular and evolutionary analyses were conducted using MEGA version 7 [55]. Comparative sequence analyses were made using available ribosomal and mitochondrial sequences of *Ae. albopictus* downloaded from GenBank.

DnaSP v.5.10.01 [56] was used to evaluate the number of haplotypes (*H*), haplotype diversity (Hd), nucleotide diversity, expressed as the average number of nucleotide differences between two sequences by site (*π*), average number of nucleotide differences between sequences (*k*), number of polymorphisms and insertions/deletions (*S*). Deviations from selective neutrality were analysed using the statistics Fu’s *Fs* and Tajima’s *D* of the neutrality test by means of the DnaSP software. In *F*_*S*_, a negative value is evidence for an excess number of alleles, as would be expected from a recent population expansion or from genetic hitchhiking, whereas a positive value is evidence for a deficiency of alleles, as would be expected from a recent population bottleneck. In Tajima’s *D*, a negative value signifies an excess of low frequency polymorphisms relative to expectation, whereas a positive value signifies low levels of both low and high frequency polymorphisms. Hierarchical analysis of molecular variance (AMOVA) among the ITS-2 of south-western Europe and Brazil samples were computed in GenALEx 6.5 [57] according to the three hierarchical levels: (i) within individuals (intra-individual); (ii) among individuals within populations (intra-population); (iii) among populations (inter-population).

#### Phylogenetic networks

Haplotype networks were generated to depict relationship among *Ae. albopictus* populations from different geographical regions with PopART [58] and Network (10.1.0.0) (http://www.fluxus-engineering.com/) software. Networks were constructed with the sequences obtained from each one of the molecular markers, using the TCS inference method in PopART, and using median-joining (MJ) network algorithm, with default parameters (equal character weight 10, transitions/transversions weight 1:1, and connection cost as a criterion) in Network. Haplotypes were connected when the parsimony had a probability of at least 0.95 of being true, as established in the theory of coalescence, starting with the shortest distance to join all the haplotypes and until the distance exceeds the parsimony limit. Hypothetical median vectors (a hypothesized sequence that is required to connect existing sequences within the network with maximum parsimony) were added to the network for shortest connection between the data set.

### Wing geometric morphometrics

Of the 111 specimens molecularly analysed, male and female mosquitoes with at least one suitable wing for morphometric analyses (Spain: 58 specimens, France: 11; Brazil: 34), were selected for phenotyping (Table 1). Wing geometric morphometrics was carried out to determine differences between *Ae. albopictus* populations. Both, the left and right wing of each specimen were dissected and mounted using STARMount® medium (Corinto Polymer Valencia, Spain) on standard microscope slides. All wings were photographed with a Nikon Digital Sight DS-L1 (Nikon Corporation) camera linked to a Nikon ECLIPSE E800 microscope. A total of 103 wing pictures of the seven populations of *Ae. albopictus* was analysed for landmark and outline-based techniques. Left wings were used unless damaged, in which case right wings were used instead. For *Ae. albopictus* phenotyping, populations were divided in males and females. Furthermore, in order to improve the sample size, populations were analysed according to the following grouping criteria: Brazil (Goiania, Jurujuba and Manaus), continental Spain (Barcelona and Valencia), insular Spain (Mallorca, Balearic Archipelago), and France (Perpignan).

#### Metric data processing

Two different approaches were applied in order to carry out a comparative morphometric analysis: landmark-based and outline-based methods.

##### Landmark-based method

**Fig. 1:**
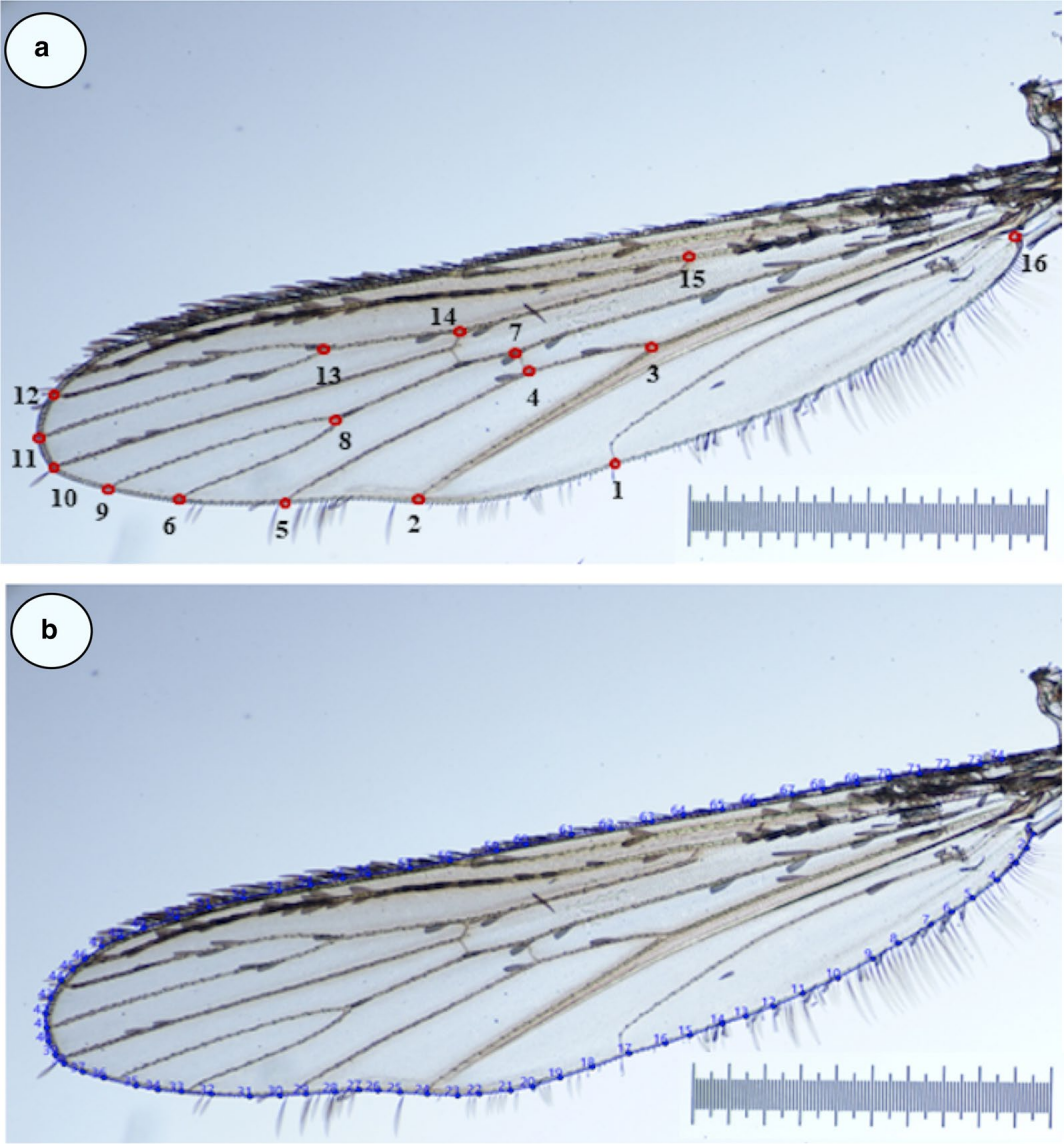
**a** Position of 16 landmarks and **b** contour digitized on *Ae. albopictus* wings for landmark-based and outline-based geometric morphometric analysis, respectively. Scale = 1 mm

Landmarks are anatomical points that can clearly be recognized among individuals and are always digitalized in the same order. Sixteen landmarks were digitalized (Fig. 1a) as previously described [59]. The “centroid size” (CS) was calculated for wing size assessment; it is defined as the square root of the sum of the squared distances between the centre of the configuration of landmarks and each individual landmark [60].

In order to analyse differences in landmarks among geographical populations, procrustes superimposition was performed, followed by centroid size (CS) comparison and discriminant analysis on partial warps or shape variables, using the MOG module of the CLIC package version 99 [61, 62], which is freely available at http://mome-clic.com.

##### Outline-based method

The outline considered for population comparisons was the external contour of the wing (Fig. 1b). Wing size was considered as the perimeter of the contour and compared between species using non-parametric tests (CS). The FOG module of the CLIC package was used to perform the Elliptic Fourier Analysis (EFA) of outline contours for the construction of shape variables [63]. Briefly, the observed contour is decomposed in terms of sine and cosine curves of successive frequencies called harmonics, and each harmonic is described by four coefficients. With this method, the first harmonic ellipse parameters are used to normalize the elliptic Fourier coefficients so that they are invariant to size, rotation, and the starting position of the outline trace. By doing this, the three first coefficients become constant (1, 0 and 0) and are not used in the remaining analyses. The fourth coefficient, the one related to the width-on-length ratio of the outline, has been used in our study. The EFA algorithm does not require the points to be equidistant [59, 64].

In order to perform a shape-based discrimination analysis, the same FOG module was used. Each pairwise Mahalanobis distance was computed between the normalized elliptic Fourier coefficients [65].

## Results

### mtDNA *cox*1 sequence analysis

The 111 *cox*1 gene sequences obtained provided 12 different haplotypes among all localities from south-western Europe and Brazil. Seven of them, were new haplotypes for *Ae. albopictus* and the other five haplotypes were detected shared with other countries, mainly with USA and Italy (Additional file 1: Table S1). These 12 haplotypes were ascribed to the codes *cox*1-H1 to *cox*1-H12 and deposited in GenBank (Acc. Nos. MW279068-MW279079). Their length was of 1433 bp, with a biased AT content of 69.7–70.1% (mean, 70.0%). The haplotype *Ae. albopictus cox*1-H1 was the most abundant, present in Valencia, Barcelona, and Mallorca Island, Spain, and in Perpignan, France. In the three distant localities analysed from Brazil (Goiás, Rio de Janeiro, and Amazonas States), only one haplotype was detected (*cox*1-H12), which was not present in south-western Europe, but already reported in Brazil (KX383924, [33]) and in Los Angeles, USA (KZ690940, [51]).

In a 1433-bp-long alignment, including the 12 *cox*1 haplotypes of *Ae. albopictus*, a total of 13 variable positions were detected, including 10 parsimony informative (p-info) positions and 3 singleton sites. Therefore, a comparative sequence analysis was made including these 12 *Ae. albopictus* haplotypes and other *cox*1 haplotypes and isolates of similar length (complete or almost complete *cox*1 gene) of the same species available in the GenBank database showing highly similar sequences according to BLASTN (Additional file 1: Table S1). This alignment, including 153 sequences (42 from GenBank and 111 from present paper), was 1433 bp-long, and contains 30 variable positions, of which, 16 p-info and 14 singleton sites, and allow us to distinguish 27 different *cox*1 haplotypes (*H*) (Table 2). When comparing the sequences of these 27 haplotypes, the pairwise *cox*1 distance matrix obtained with PAUP shows that the total number of absolute differences and Kimura 2-parameter distances ranged between 1 to 12 (average 4.98) and 0.0007 to 0.0083 (0.003), respectively. The highest values were obtained when comparing haplotypes from Philippines with haplotypes present in other countries (Additional file 2: Table S2).

**Table 2:**
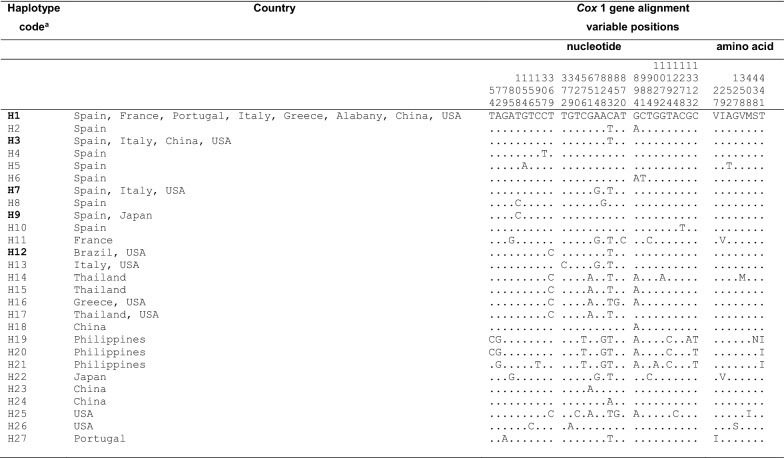
Nucleotide and amino acid differences found in the mtDNA *cox*1 gene sequence of *Ae. albopictus* populations studied and other haplotypes and isolates of the same species from GenBank

Numbers (to be read in vertical) refer to variable positions obtained in the alignment made with MEGA 7;. = identical

^a^ Haplotypes H1 to H12 correspond to to *Ae. albopictus cox*1-H1 to *cox*1-H12 from present paper (five of them in bold, shared with other countries). H13 to H27 correspond to haplotypes retrieved from GenBank (see Additional file 1: Table S1)

COX1 protein provided 3 haplotypes for all samples analysed from Brazil and south-western Europe, each other differing by only one amino acid position. Nucleotide and amino acid differences with *Ae. albopictus* haplotypes and other isolates are detailed in Table 2.

### *cox*1 genetic diversity and haplotype networks

In a first step, sequences obtained from 111 specimens, representing the 12 haplotypes here described from south-western Europe and Brazil, were analysed. The global haplotype diversity (Hd) was 0.752 (SD = 0.025), the nucleotide diversity (*π*) was 0.00108 (SD = 0.00009), the average number of nucleotide differences was 1.544, and the number of polymorphisms was 13. Tajima’s *D* and Fu’s *Fs* values were − 0.9953 (*P* > 0.10) and = − 3.363, respectively. A detailed analysis of the genetic diversity of each of the populations analysed is noted in Table 3a.

**Table 3:** Genetic diversity of *Ae. albopictus* populations from south-western Europe and Brazil. (a) Based on mtDNA *cox*1 gene sequences;( b) based on 5.8S-ITS-2 rDNA sequences

(a)									
Geographical origin	*n*	*H*	Variable sites	Mutations (P-info + singleton sites)	*K*	Hd ± SD	Nd ± SD (× 10^–2^)	Tajima’s *D*	Fu’s *Fs*
Valencia	26	5	5	2 + 3	0.59	0.406 ± 0.116	0.042 ± 0.015	− 1.556 (*P* > 0.10)	− 2.192
Barcelona	14	4	3	2 + 1	0.87	0.495 ± 0.151	0.061 ± 0.020	− 0.244 (*P* > 0.10)	− 0.728
Mallorca Island	25	6	5	4 + 1	1.55	0.723 ± 0.073	0.110 ± 0.016	0.4958 (*P* > 0.10)	− 0.619
Perpignan	10	2	5	5 + 0	1.94	0.389 ± 0.164	0.137 ± 0.058	0.2412 (*P* > 0.10)	3.702
Goiania, Jurujuba Manaus	36	1	–	–	–	–	–	–	–

(b)										
Geographical origin	*n*^a^	*H*	Variable sites^b^	Mutations (P-info + singleton sites)	indels	*K*	Hd ± SD	Nd ± SD (× 10^–2^)	Tajima’s *D*	Fu’s *Fs*
Valencia	46	34	76	17 + 24	35	3.50	0.905 ± 0.038	0.773 ± 0.119	− 2.155 (*P* < 0.05)	− 25.061
Barcelona	17	16	32	6 + 15	11	4.22	0.971 ± 0.032	0.885 ± 0.141	− 1.273 (*P* > 0.10)	− 7.968
Mallorca Island	45	33	103	13 + 24	66	4.73	0.947 ± 0.022	1.120 ± 0.115	− 1.620 (0.10 > *P* > 0.05)	− 18.034
Perpignan	41	30	115	14 + 18	83	3.59	0.937 ± 0.023	0.885 ± 0.104	− 1.897 (*P* < 0.05)	− 12.790
Goiania,	50	38	87	17 + 30	40	7.46	0.963 ± 0.015	1.658 ± 0.081	− 1.144 (*P* > 0.10)	− 17.039
Jurujuba	26	14	83	13 + 3	67	4.56	0.862 ± 0.045	1.078 ± 0.115	0.306 (*P* > 0.10)	− 0.319
Manaus	28	22	78	14 + 18	46	7.17	0.968 ± 0.024	1.612 ± 0.105	− 0.660 (*P* > 0.10)	− 10.540

*n*, samples sequenced; *n*^a^, sequences obtained (cloning + sequencing); ^b^, including mutations + sites with alignment gaps. *p* values are indicated in parentheses

*H*, *n*º of haplotypes; P-info, parsimony informative; Indels, insertions/deletions; *K*, average number of nucleotide differences; Hd, haplotype (gene) diversity; Nd, nucleotide diversity (per site)

**Fig. 2:**
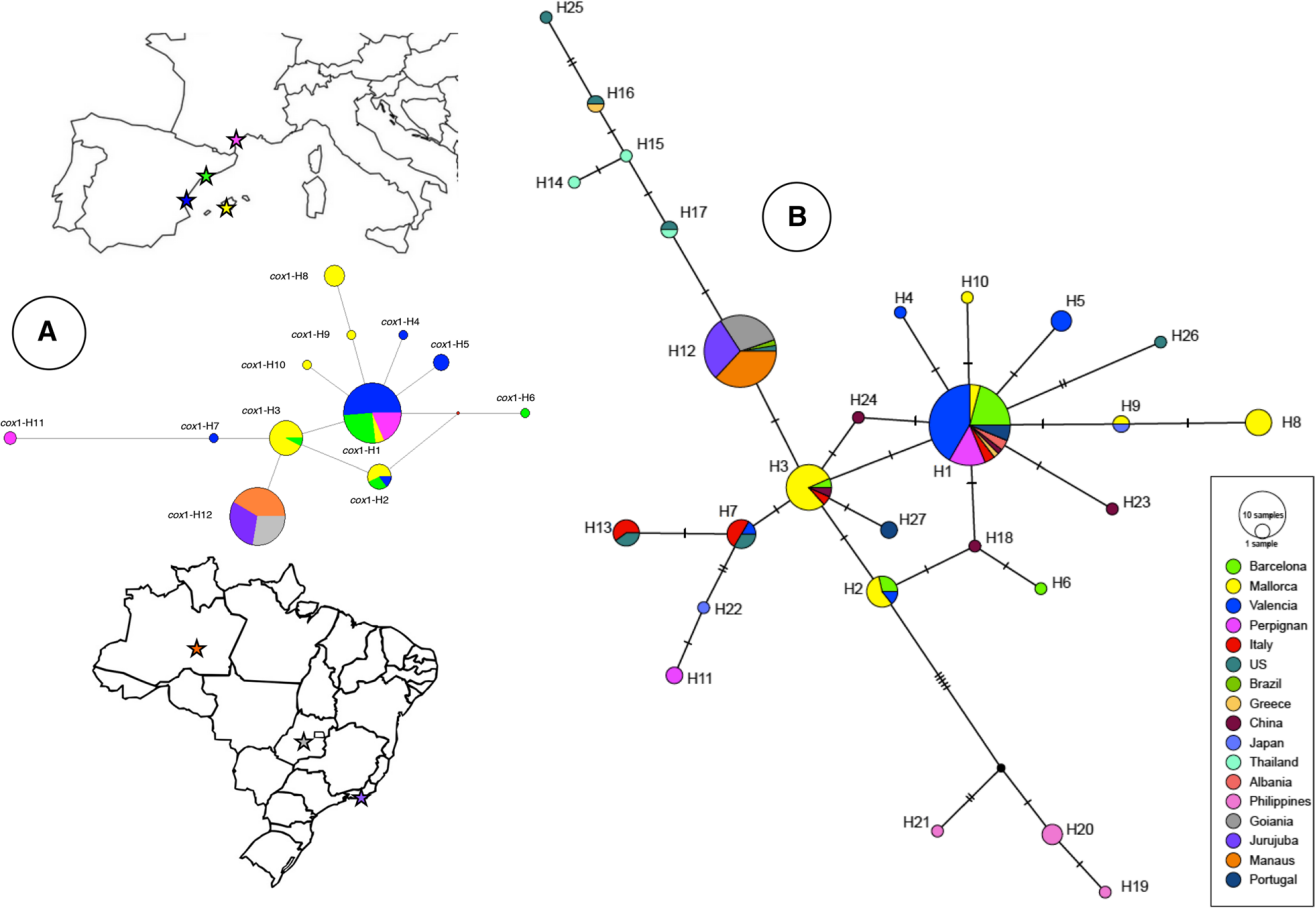
Phylogenetic networks of *Ae. albopictus* haplotypes based on mtDNA *cox*1 sequences. **a** Median network with south-western Europe and Brazil populations. The area of each haplotype is proportional to the total sample. Small red-filled circle represents intermediate haplotype not present in the sample. Mutational steps between haplotypes are represented by line length. **b** TCS network with different localities and countries in Asia, Europe, and the Americas. Circles are proportional to the number of samples represented for each haplotype. Slashes on branches between nodes indicated mutations. The colors represented in the legend correspond with those represented by the haplotype network. Haplotype information detailed in Additional file 1: Table S1

The median joining haplotype network allows us to distinguish the clustering of all samples from Brazil in only one haplotype (cox1-H12), distant by only one mutational difference from haplotype cox1-H3, present in Barcelona and Mallorca (Fig. 2a). Samples from France include two haplotypes: the cox1-H1, the most common haplotype; and the cox1-H11, to which two French specimens proved to belong. This haplotype cox1-H11 is not shared with any other sample and appears distant from the other European and the Brazilian haplotypes in the network. Samples from Mallorca Island split into 6 haplotypes, three of them shared with no other population and thus representing the locality with the highest number of haplotypes, followed by Valencia (5 haplotypes), Barcelona (4) and Perpignan (2) (Fig. 2a, Additional file 1: Table S1). The most common haplotype was cox1-H1, which comprises samples from all southern European localities studied, including continental and insular Spain and France (Fig. 2a).

A second network analysis was performed including 153 specimens representing our 12 haplotypes (*cox*1-H1 to *cox*1-H12), plus 42 sequences representing haplotypes/isolates from different localities and countries in Asia, Europe, and the Americas (haplotypes H13 to H27) (Additional file 1: Table S1). This *cox*1 haplotype network comprises twenty seven haplotypes. Both TCS and median-joining networks showed the same topology, and consequently only the TCS network is shown (Fig. 2b). Two central haplotypes in the network concern the majority of specimens and countries. The haplotype *cox*1-H1 includes samples from all the Spanish localities and France (Perpignan) and proves to be the most common haplotype, present in 9 countries (Italy, Greece, Albany, Spain, Portugal, France, USA, China and Japan) (Fig. 2b, Additional file 1: Table S1). The haplotype *cox*1-H3 is also highly distributed, not only in Spain (Barcelona and Mallorca), but also in Italy, China, and USA. The haplotype network shows the *cox*1-H1 haplotype to be highly connected, in a star distribution, by only one mutational step of distance with many haplotypes from Spain, but also with some ones from China (H23, H24, H18) and, at two mutational steps, with one haplotype from USA (H26).

The *cox*1-H3 haplotype is linked to haplotypes from Thailand, Greece and USA. The French haplotype *cox*1-H11 appears far away from other European haplotypes in the network, but interestingly linked to haplotype H22 from Japan. Finally, Philippines haplotypes appear clustering together, but are the most distant from haplotypes from other countries (Fig. 2b). No haplotype-groups became apparent, with only a core group including all European haplotypes (with the exception of one haplotype from Greece, H16), plus several haplotypes from Asia and USA, some of them shared with European haplotypes. In summary, from our 12 haplotypes here described, six are exclusive for Spain (*cox*1-H2, *cox*1-H4, *cox*1-H5, *cox*1-H6, *cox*1-H8, *cox*1-H10), one exclusive for France (*cox*1-H11), and five (*cox*1-H1, *cox*1-H3, *cox*1-H7, *cox*1-H11, *cox*1-H12) are shared with other countries, mainly with Italy, USA and China. It is worth mentioning that the European haplotype more linked to Asiatic haplotypes is *cox*1-H2, present only in Barcelona and Mallorca Island (Fig. 2b).

### rDNA ITS-2 sequence analysis

The rDNA sequence fragment obtained included an 80-bp-long fragment of the 5.8S gene, followed by the complete ITS-2 of a length of 349–407 bp (average 401.1 bp) and subsequently a small last fragment of the initial at least 20 nucleotides of the 28S. After deleting the small 28S fragment, a total of 253 such 5.8S-ITS-2 sequences were obtained from the mosquitoes analysed. These sequences collapsed into 162 haplotypes (GenBank Acc. Nos. MW281865-MW282026).

The pronounced length variability of the ITS-2 was assessed by means of the alignment of these 162 haplotypes. This alignment was 491-bp-long and contained 337 conserved positions and 153 variables sites, of which, 50 were p-info and 103 singleton sites. The ITS-2 length variability was seen to be caused by regions of insertions in consecutive positions which are unexpectedly long, i.e. concerning more than 10 consecutive positions. Such long regions involved 57–58 and/or 20–30 consecutive insertion/deletions (indels) located in the positions 302–361 and 333–389 of the total alignment, respectively. These correspond to positions 222–281 and 253–309 of the ITS-2. In Mallorca and Perpignan, two and three clone sequences showed such deletion regions in the alignment, respectively. In Manaus, such deletion regions were observed in up to six clone sequences. Only in Barcelona, in the aforementioned alignment no such clone sequence showing long deletion regions was detected in the long-sized ITS-2 of the specimens from this origin.

The length and GC content of the 162 haplotypes of the 5.8S-ITS-2 rDNA varied between 429 and 487 bp in length (481.2 bp) and 52.8–56.8% (55.5%), respectively. Sequences were aligned according to their geographical locations and polymorphic sites were analysed. All populations presented some mutated positions in the conserved fragment of the 5.8S rRNA gene, ranging from 0–7 mutations (average 2.14), except in samples from Barcelona, whereas in the ITS-2 mutations ranged between 16 and 47 (36.9). Except in Jurujuba, in all populations the majority of these mutations were due to singleton sites. Gaped positions, generated by indels, were observed in the alignments of each population, ranging from 11–83, with an average of 49.2 indels (Table 3b).

The large number of haplotypes found within a same individual is noteworthy, ranging 2–7 haplotypes (average, 4.7) and 1–6 (3.3) in European and Brazilian specimens, respectively. The number of haplotypes found in a population was also very high, ranging 16–34 (27.6) haplotypes in Spain (continental and insular), 30 haplotypes in France, and 14–38 (24.7) in Brazil.

When comparing the sequences of these 162 haplotypes, the pairwise 5.8S-ITS-2 evolutionary divergence matrix obtained with MEGA 7 shows that the total number of absolute differences (gaps not included) in pairwise comparisons ranged between 1 and 14 (average 4.8). The highest values were obtained when comparing haplotypes from Valencia with haplotypes from Manaus (Table not shown).

A comparative ITS-2 sequence analysis was made including our 162 *Ae. albopictus* haplotypes and other haplotypes of similar length (complete or almost complete ITS-2) of the same species available in the GenBank and showing highly similar sequences according to BLASTN. The first data set included 258 sequences, of which 162 from the present study and 96 from GenBank (representing haplotypes from Italy, Greece, Reunion Island, Thailand, Vietnam, China, and USA). Among these 258 sequences, haplotypes of *Ae. albopictus *sensu stricto and *Aedes* sp. cryptic species [37, 66] were included. Sequence analysis comparison allowed us to confirm that no haplotypes corresponding to the subgroup of *Ae. albopictus* cryptic species were present in our samples.

**Fig. 3:**
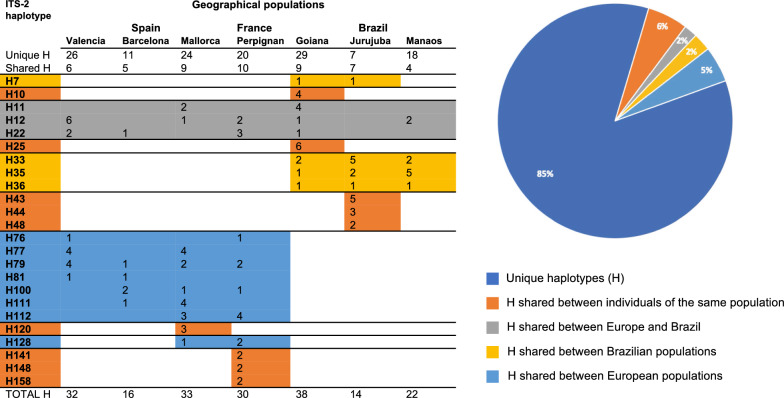
Numerical and graphical distribution of *Ae. albopictus* 5.8S-ITS-2 rDNA unique and shared haplotypes between countries and populations according to DnaSP, *H* haplotype

A second analysis was performed retrieving those cryptic haplotypes. The data set resulted in 230 sequences, 162 from our samples plus 68 from GenBank, representing the same seven aforementioned countries. Due to differences in length among the samples included in this data set, the region corresponding to the 5.8S gene was deleted and sequences were adjusted to the same length of the ITS-2 in a 421 bp-long alignment. This alignment contains 244 variable positions of which, 117 were p-info and 127 singleton sites. These 230 sequences collapsed into 185 haplotypes according to ALTER. Among these 185 haplotypes, only 12 GenBank entries showed identity with 8 of our haplotypes. Italy was the country sharing most haplotypes (6) with Valencia (H89), France (H151), Goiania (H12, H16, H38), and Manaus (H49). Two of the haplotypes shared between our European and Brazilian populations (H12 and H25) (Fig. 3) were also detected in Italy, Thailand, and USA (Table 4).

**Table 4:** European and Brazilian *Ae. albopictus* ITS-2 rDNA haplotypes shared with other countries

Country (locality)	Shared haplotypes		Country (locality)	GenBank Acc.Nº	References
	ITS-2 haplotype	Haplotype/isolate			
Spain (Valencia)	H89	Hap22	Italy (Pavia)	KY703689	[38]
Spain (Valencia)	H94	isolate KF7	China (Henan)	MF623839	[65]
France (Perpignan)	H151	Hap18/Aa18	Italy (Pavia)	KY703675	[38]
		Hap49/Aa49	France, Reunion St. Pierre	KY703676	[38]
Brazil (Goiania)	H12	–	USA	M95127	[86]
		Hap13/Aa13	Thailand (Phato)	KY703691	[38]
		Hap23/Aa23	Italy (Pavia)	KY703692	[38]
Brazil (Goiania)	H16	Hap44/Aa44	Italy (Modena)	KY703688	[38]
		H3	Vietnam (Bin Phuoc)	KX495935	[37]
Brazil (Goiania)	H25	Hap09/Aa9	Thailand (Phato)	KY703695	[38]
Brazil (Goiania)	H38	Hap21/Aa21	Italy (Pavia)	KY703685	[38]
Brazil (Manaus)	H49	Hap42/Aa42	Italy (Cesena)	KY703669	[38]

Sequences adjusted to the same length of the ITS-2 in a 421 bp long alignment

H or Hap, Haplotype

Special attention was paid to our shorter ITS-2 sequences, that is, those presenting a large consecutive region of deletions. We only found one haplotype (Aa45—KY03659) from St. Denis in Reunion Island [39], presenting a large region of consecutive deletions (77) and located between the similar alignment positions (319 to 398) as we found in our samples.

When comparing the sequences of the 253 specimens representing these 162 haplotypes, the pairwise 5.8S-ITS-2 distance matrix obtained with MEGA 7 allowed us to analyse the overall mean distance at intra-individual, intra- and inter-populational groups, showing that the total number of differences (ts + tv) ranged 3.3–6.0 (average, 4.7), 3.7–7.5 (5.6) and 4.6–8.9 (6.7), respectively. Substitutions per site (using Kimura 2-parameter model) ranged between 0.00964 and 0.01919 (average 0.01433) between the seven populations. The highest values were obtained when comparing intraindividual sequences and intrapopulation sequences from Goiania. At inter-population level, the greatest genetic distance (8.9) was obtained between Manaus and Jurujuba, while the shortest (4.6) were between Valencia, Barcelona and Perpignan populations (Table 5). However, when the tropical populations of Brazil were compared with those of temperate zones of Europe, this genetic distance was 7.5, slightly lower than among Brazilian populations.

**Table 5:** Pairwise distances between 5.8S-ITS2 sequences of *Ae. albopictus* samples according to MEGA 7

	Estimates of average evolutionary divergence over all sequence pairs								
	Overall mean distances		Goiania	Jurujuba	Manaus	Valencia	Barcelona	Mallorca	Perpignan
	Intraindividual	Intra-population							
Goiania	6.0	7.5		0.01882	0.01693	0.01554	0.01583	0.01575	0.01570
Jurujuba	5.9	5.7	8.8		0.01919	0.01679	0.01600	0.01800	0.01638
Manaus	5.6	7.2	7.9	8.9		0.01518	0.01539	0.01608	0.01508
Valencia	3.3	3.7	7.3	7.9	7.1		0.00964	0.01062	0.00970
Barcelona	3.6	4.7	7.5	7.6	7.2	4.6		0.01177	0.01064
Mallorca	5.0	5.6	7.4	8.5	7.5	5.0	5.6		0.01205
Perpignan	3.6	4.9	7.4	7.7	7.0	4.6	5.0	5.7	

Below diagonal, overall mean distances estimated using total number of differences (ts + tv); above diagonal, base substitutions per site using the Kimura 2-parameter model

The analysis of molecular variance (AMOVA) within and among *Ae. albopictus* populations is detailed in Table 6. Most of the variation occurs within individuals (73%), while 9% and 18% of the total variation occurs among populations and among individuals within populations, respectively. Percentage of variation among the 3 geographical groups (Spain, France and Brazil) were only 4%.

**Table 6:** Hierarchical analysis of molecular variance (AMOVA) within and among *Ae. albopictus* populations using 5.8S-ITS2 sequence data. (a) Comparing seven populations: Valencia, Barcelona, Mallorca, Perpignan, Goiania, Jurujuba and Manaus; (b) comparing three geographical populations groups: Spain, France and Brazil

Source of variation	d*f*	Sum of squares	Estimates of variances	Percentage of variation
(a) Among populations	6	148.088	0.458	9
Among individuals within populations	48	369.059	0.895	18
Within individuals	198	725.564	3.664	73
Total	252	1242.711	5.017	100
(b) Among groups	2	76.619	0.224	4
Among population within groups	4	71.469	0.404	8
Within populations	246	1094.623	4.450	88
Total	252	1242.711	5.078	100

d*f*, degrees of freedom

### ITS-2 genetic diversity and haplotype networks

Haplotype network analysis revealed 162 unique haplotypes among the 253 sequences obtained from south-western Europe and Brazil**.** The global haplotype (gene) diversity (Hd) was 0.927 (SD = 0.013), the nucleotide diversity (*π*) was 0.01059 (SD = 0.00049), the average number of nucleotide differences was 4.225, and the number of polymorphisms was 153. Tajima’s *D* and Fu’s *Fs* values were − 2.422 (**, *P* < 0.01) and = − 180.685, respectively. At population level, the Hd estimates across the analysed sequences ranged from 0.862 ± 0.045 in Jurujuba (Brazil) to 0.971 ± 0.032 in Barcelona (Spain), and the nucleotide diversity (*π*) ranged between 0.00773 ± 0.00119 in Valencia (Spain) up to 0.01658 ± 0.00081 in Goiania (Brazil). A detailed analysis of the genetic diversity of each population analysed is listed in Table 3b. In all populations except in Jurujuba, the majority of polymorphisms (gaps not considered) were caused by single base variation.

**Fig. 4:**
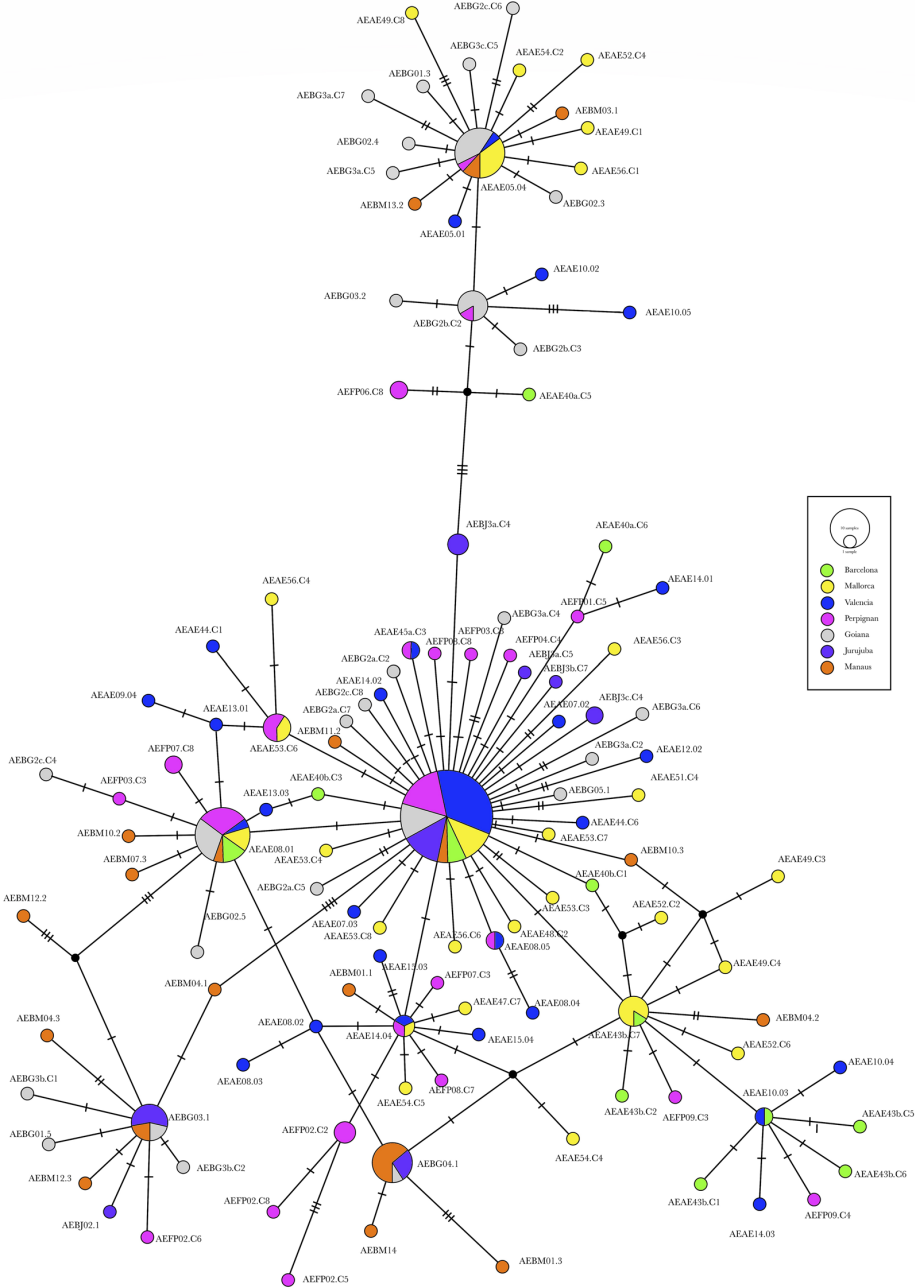
TCS network among identified 5.8S-ITS-2 haplotypes of *Ae. albopictus* from south-western Europe and Brazil according to PopART. Circles are proportional to the number of samples represented for each haplotype. Slashes on branches between nodes indicated mutations. The colors represented in the legend correspond with those represented by the haplotype network. The smallest black nodes indicate unobserved haplotypes (median vectors)

The haplotype ITS2-H12 was the most common, including samples from Goiania and Manaus (Brazil), Valencia and Mallorca (Spain) and Perpignan (France). A large portion of the haplotypes, namely 138/162 (mean 85.2%), proved to be unique, whereas 9/162 (5.6%) were shared only between specimens of the same population, and 15/162 (9.2%) were shared among populations. Among those 15 haplotypes, 3 (1.85%) were shared between Brazilian and European populations, 8 (4.93%) were shared exclusively between Mediterranean populations of Europe (Valencia, Barcelona, Mallorca and Perpignan), and the remaining 4 (2.46%) haplotypes appeared shared between only Brazilian populations (Fig. 3). The TCS network generated by PopART illustrated the relationships between the 162 haplotypes. No haplotype-groups were apparent according to geography, with all haplotypes appearing connected to each other by no more than 3 nucleotide substitutions (Fig. 4).

### Landmark-based geometric morphometrics

**Fig. 5:**
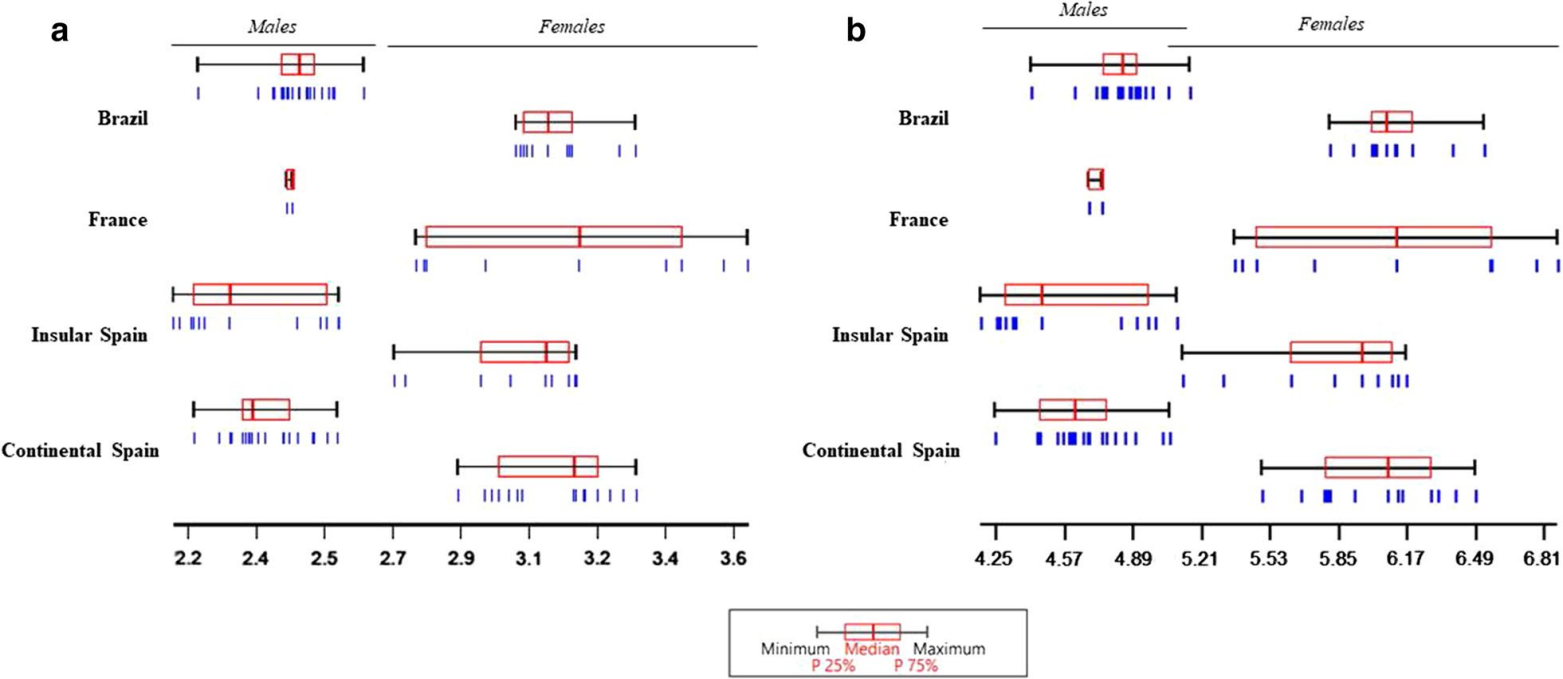
**a** Variation of centroid size and **b** wing perimeter (converted from pixels to mm) of females and males of *Ae. albopictus* from south-western Europe and Brazil. Each box shows the group median that separates the 25th and 75th quartiles and range. Each blue bar represents one specimen

As shown in Fig. 5a, mean wing CS comparison presented consistent size variation between sexes but not within groups, i.e. females had larger wings than males in all the populations analyzed. In the females, specimens from France had the largest wings, followed by continental Spain, Brazil and insular Spain. In the males, specimens from Brazil had the largest wings, followed by France, continental Spain, and insular Spain. Summing up, Spanish insular populations had the smallest wings in both sexes (Table 7a).

**Table 7:** Phenotypic diversity of *Ae. albopictus* from south-western Europe and Brazil. (a) Landmark-based (LB) and outline based (OB) Mahalanobis distances (LB/OB) obtained with discriminant analysis between wing shapes of female (left) and male (right); (b) comparative morphometric data (extreme values, mean, standard deviation and standard error) of wing centroid size and wing perimeter

(a)				
	Continental Spain	Insular Spain	France	Brazil
Males				
Continental Spain	0.00			
Insular Spain	4.38^a^/9.34	0.00		
France	6.51/15.07	5.90/16.12	0.00	
Brazil	4.03^a^/12.27	4.61^a^/8.94	6.63/21.12	0.00
Females				
Continental Spain	0.00			
Insular Spain	4.99/9.80	0.00		
France	4.00/8.30	4.04/6.90	0.00	
Brazil	4.41/6.70	5.95/9.57	5.38/9.87	0.00

(b)						
Population	Sex	*N*	Centroid size Mean ± S.D. (Min–Max) (mm)	Centroid size S.E	Wing perimeter Mean ± S.D. (Min–Max) (mm)	Wing perimeter S.E
Continental Spain	Female	15	3.09 ± 0.13 (2.87–3.30)	0.03	6.09 ± 0.28 (5.56–6.55)	0.07
	Male	22	2.42 ± 0.09 (2.25–2.59)	0.02	4.78 ± 0.19 (4.38–5.17)	0.04
Insular Spain	Female	9	3.00 ± 0.17 (2.72–3.15)	0.06	5.89 ± 0.36 (5.24–6.23)	0.12
	Male	12	2.38 ± 0.16 (2.20–2.59)	0.05	4.71 ± 0.33 (4.33–5.19)	0.10
France	Female	9	3.14 ± 0.32 (2.77–3.56)	0.11	6.16 ± 0.58 (5.50–6.89)	0.19
	Male	2	2.48 ± 0.01 (2.47–2.48)	0.01	4.85 ± 0.06 (4.81–4.89)	0.04
Brazil	Female	11	3.11 ± 0.10 (3.01–3.29)	0.03	6.18 ± 0.19 (5.89–6.56)	0.06
	Male	23	2.49 ± 0.08 (2.26–2.65)	0.02	4.96 ± 0.14 (4.53–5.26)	0.03

^a^Statistically significant pairwise Mahalanobis distances; *N*, sample size; S.D., standard deviation; Min, minimum; Max, maximum; mm, millimeter; S.E., standard error

**Fig. 6:**
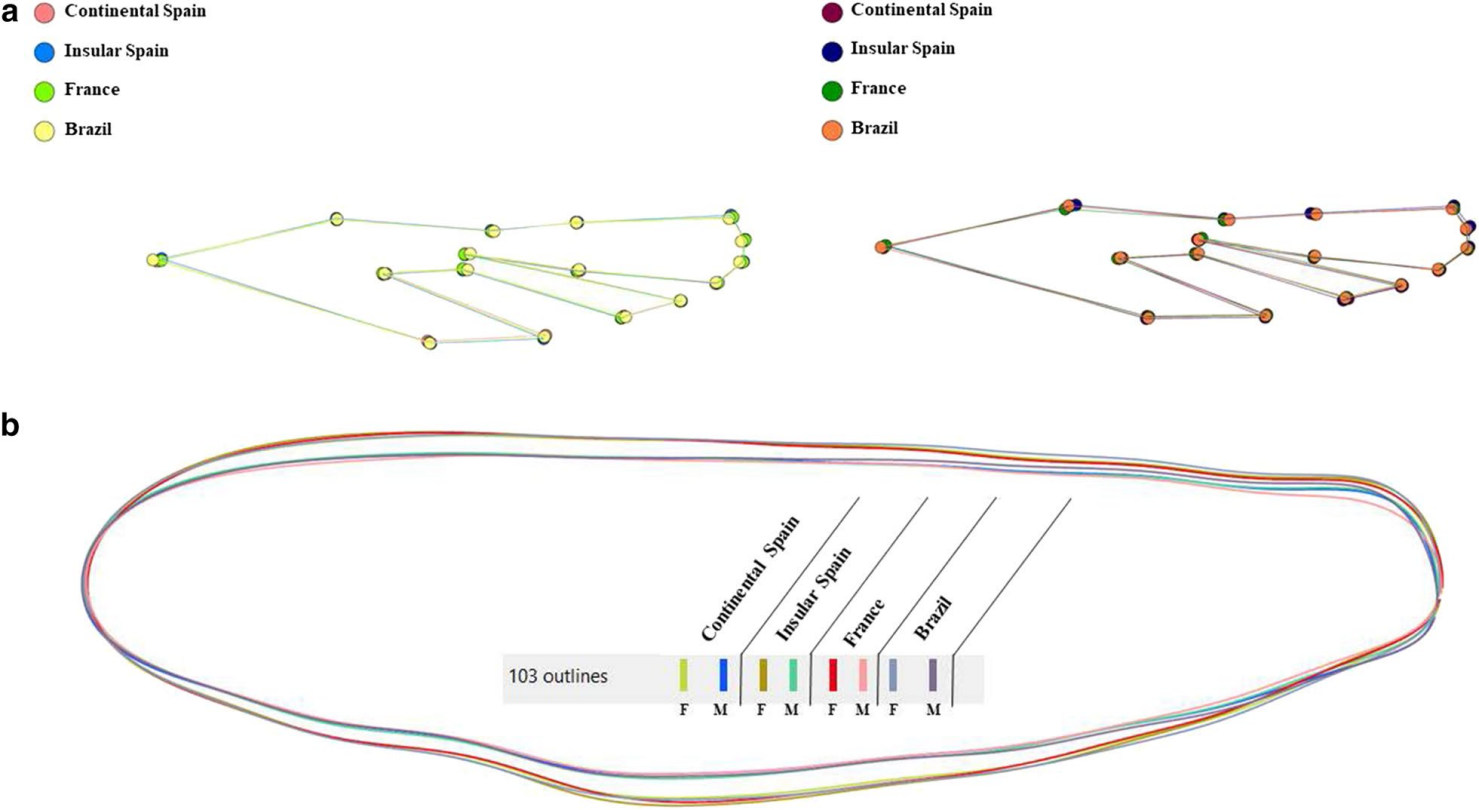
**a** Superposition of the mean landmark configurations and **b** outlines of *Ae. Albopictus* from south-western Europe and Brazil. Left, females (*F*); right, males (*M*)

**Fig. 7:**
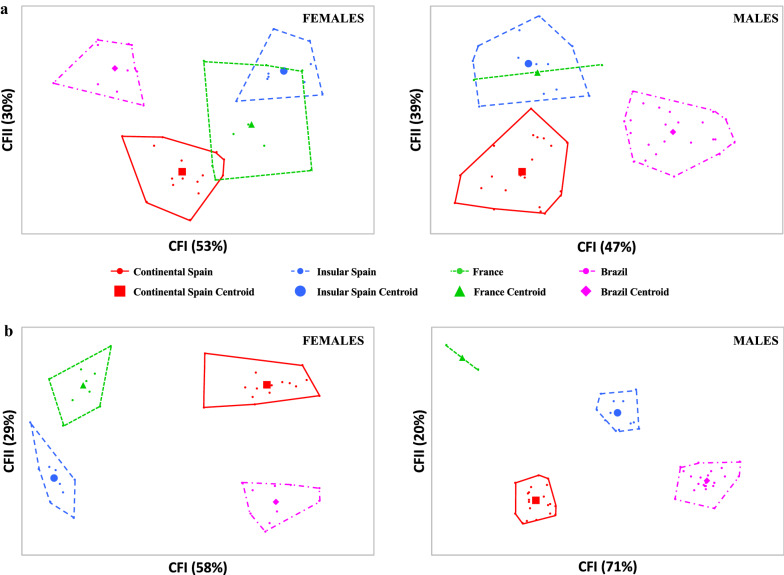
**a** Landmark-based and **b** outline-based discriminant analysis of *Ae. albopictus* from south-western Europe and Brazil. Factor map of the two canonical factors (*CFs*) derived from final shape variables for females (left) and males (right). Each point represents a specimen. The horizontal axis is the first CF; the vertical axis is the second CF. In brackets, percentage of contribution

Landmark superposition (without any amplification) did not show noticeable differences. However, some landmark displacements were visible in the final part of the female wings and in the upper and the final part of the male wings (Fig. 6a). In both sexes, there were differences in wing shape, although significant differences between populations were only found in males, in the case of specimens from continental Spain *vs* insular Spain and insular Spain *vs* Brazil (Table 7a). Landmark-based discriminant analysis showed a separation between European and Brazilian populations, in both males and females. Furthermore, landmark-based discriminant analysis showed an overlap in European groups in both males and females (Fig. 7a). Summing up, the greatest differences in wing shape were detected when insular Spanish vs European and Brazilian male specimens were compared. Discriminant analysis of wing shape variables showed a clear separation between European and Brazilian specimens in both males and females (Fig. 7a).

### Outline-based geometric morphometrics

Size was considered as the outline perimeter and yielded similar results to those obtained in the CS analysis (Table 7b). The correlation was excellent between the outline perimeter and CS, with scores above 0.99 (detailed results not shown). Direct visualization, without amplification, of contour differences showed wider and longer wings for females than for males in all populations (Fig. 6b). Females and males from Brazil presented the largest wings, followed by France, continental Spain and insular Spain (Fig. 5b, Table 7b). Summing up, in both females and males, European insular specimens present a smaller wing perimeter *vs* European and Brazilian specimens.

For the outline-based method, the factor maps of the discriminant analysis (Fig. 7b) showed a separation of all groups analyzed, in males as well as in females. Pairwise comparisons of Mahalanobis distances showed the greatest shape differences between groups from Brazil and France in both males and females (Table 7b). It is noteworthy that distances were generally longer between males than between females. Summing up, outline-based discriminant analysis showed differences in all groups analyzed from the different geographical areas, with European and Brazilian specimens presenting the greatest shape differences in both male and female specimens. A detailed pairwise comparison between wing shapes of females and males of the 7 populations studied using landmark-based (LB) and outline based (OB) Mahalanobis distances (LB/OB) is shown in Additional file 3: Table S3.

## Discussion

This is the first study that explores the combined use of rDNA/mtDNA and phenotypic markers to assess *Ae. albopictus* diversity and relationships in south-western Europe and Brazil. In Europe, the climate is temperate, and the introduction of this mosquito is recent (first record in 1979 in Albania—[67]), including both established and not yet established populations. In Brazil, the climate of the selected populations is tropical (States of Amazonas, Rio de Janeiro, and Goias) and the introduction dates from 1986 [68]. In both cases, a fast spread has been reported in recent years.

### mtDNA marker analyses

Regarding the *cox*1 marker, different haplotype variability results have been obtained in numerous studies on *Ae. albopictus* in different geographical zones [66, 69, 70], despite the short length of the *cox*1 fragments used in many of these studies [32]. According to the current trend of analysing the complete gene or the complete mitogenome [33, 39, 41, 51], we have performed the *cox*1 haplotyping based on a long fragment of 1433 bp (out of a total of 1537 bp) of populations from south-western Europe and Brazil to compare their potential genetic similitude and their phylogenetic relationships with other strains from Europe, Asia and the Americas. In this analysis, surprisingly no one *cox*1 haplotype appeared shared by Europe and Brazil, suggesting different genetic structure and possible different susceptibility to infectious agents.

Despite having studied seven populations, from three countries and two continents, the total number of *cox*1 haplotypes obtained in the present study is remarkably small (12 haplotypes), of which 6 are exclusive for Spain, 1 for France, and 5 shared (1 between Brazilian populations and 4 between European populations), and with very little genetic variability (0.90% of polymorphic sites). When this *cox*1 analysis was expanded including 153 samples representing 13 countries in Europe, Asia and the Americas, the number of haplotypes obtained was also scarce (27 haplotypes) and the genetic variability was low (2.0%). These results differ from the 66 haplotypes obtained when sequencing the same *cox*1 fragment (1433 bp) in 12 populations from several countries of 2 continents, covering a wider geographic region [51]. A lower level of *cox*1 polymorphism, or high genetic similarity, was also reported in other European populations, not allowing to resolve the structuring of the different populations [39, 71]. This situation may be attributed not only to the low level of resolution of this mtDNA marker in *Ae. albopictus*, but also to the recent invasion of this species into countries or the inclusion of samples from a relatively narrow geographical range [70]. The overall negative values of Tajima’s D and *F*_*S*_ in the neutrality test indicate an excess of low frequency polymorphisms and a recent population expansion.

The low diversity found in mtDNA markers was initially hypothesized to be the consequence of a mitochondrial hitchhiking caused by the co-infection by the two strains of the endosymbiotic bacteria *Wolbachia* found co-infecting almost all *Ae. albopictus* populations studied [32, 72]. However, it should be considered that such reduced diversity could have been a biased result due to the analysis of specimens derived from mosquito colonies. Additionally, the detection of a pronouncedly polymorphic region in the *cox*1 gene when working with longer sequences of this gene posed a question mark on the *Wolbachia* impact hypothesis [51, 73].

The median joining haplotype network of *cox*1 (Fig. 2b) shows the greater kinship of the Spanish and French haplotypes with the Asian haplotypes (haplotypes from China and Japan found in Valencia, Barcelona, Mallorca, and Perpignan). This could be explained by the growing trade and travel relationships between those Asian and European countries. Among the 12 *cox*1 haplotypes here described, five of them are shared with other countries, mainly Italy, China and USA. Our results are in agreement with other studies that report the existence of one haplotype considered the most abundant [38, 51, 66]. The haplotype *cox*1-H1 is highly represented in our samples from Spain and France as well as in Italy, Greece, Portugal, Albany, USA, China and Japan, followed by a second haplotype (*cox*1-H3) highly distributed not only in Spain (Barcelona and Mallorca), but also in Italy, China and USA.

The absence of genetic variability in the *cox*1 gene among the Brazilian populations, despite the long geographical distances between the populations studied (located in Rio de Janeiro, Goiás and Amazonas), should be emphasized. This may be due to its recent introduction and spread, as suggested by its very recent first detection in this country [27]. All of the Brazilian samples presented the same *cox*1 haplotype (cox1-H12). Reduced levels of genetic variation in *Ae. albopictus* from Manaus (Amazonas state), and in other four populations from south eastern Brazil, have been also detected, although based on the mtDNA gene coding for NADH dehydrogenase subunit 5 (*nad*5) [74, 75].

### rDNA marker analyses

The rDNA ITS-2 is considered as relatively conserved and species-specific. Among the hugely considered molecular markers for mosquito taxonomy, ITS-2 have been widely used to infer relationships at species level, up to even closely related species, subspecies or populations [29].

Although the number of ITS-2 sequences of *Ae. albopictus* is recently increasing in GenBank, few studies have focused on this marker to infer intra-individual or intra- and inter-populational variability [39]. Most of these works use ITS-2 for specific classification and molecular characterization [71, 76–80] or for the distinction of cryptic species [37, 66]. Our study is the first to provide an in-depth analysis of genetic variability of *Ae. albopictus* based on 5.8S-ITS-2 and highlights the presence of high intraspecific variability among populations collected from different eco-regions. This genetic variability was revealed by:

- differences in length, ranging from 429 to 487 bp (average 481.2 bp);
- high mutation rate (16–47; mean 36.9);
- high indel number (11–83; 49.2);
- mutations in the conserved 5.8S fragment (0–7; 2.14);
- high number of haplotypes obtained, both within the same specimen and within the same population;
- overall pairwise distances similar within individuals **(**3.3–6.0; mean 4.7) than between individuals of the same population (3.7–7.5; 5.6) and both slightly less than between populations (4.6–8.9; 6.7).

According to AMOVA, most of the genetic variation was observed within individuals (73%) or within populations (88%) and not among populations (9%) or among groups (4%) at both singe population and at geographic region groups, respectively.

The 253 5.8S-ITS-2 sequences here obtained provided 162 unique haplotypes, of which 66 haplotypes for Brazil, 73 for Spain and 23 for France. Their alignment contains 153 variable positions, which represent a variability of 31.2%, considerably greater (about 35 times higher) than those obtained with the *cox*1 (0.9%) for the same specimens and geographical origins. This high 5.8S-ITS-2 variability has allowed us to describe a high number of haplotypes within the same specimen: 2–7 haplotypes (mean 4.7) in south-western Europe, and 1–6 (3.3) in Brazil. The ITS-2 haplotype number is higher than the mean of 2 haplotypes per individual previously reported [39].

The number of ITS-2 haplotypes obtained for each one of the seven geographical populations was also high, ranging 16–34 (27.6) haplotypes in Spain (continental and insular), 31 haplotypes in France and 14–38 (24.7) in Brazil. The high number of haplotypes in the same population or the fact of sharing few haplotypes between populations, coincides with previous reports about native and introduced populations of this vector [39]. Overall, we found higher genetic pairwise distances among tropical Brazilian populations (5.7–7.5; 6.8) than among temperate European populations (3.7–5.6; 4.7), in which the considered founder colony in Spain (Barcelona) is included.

In regions of the rDNA operon with faster evolution, as is the case of ITS-2, the appearance of indels is common even in different populations of the same species. However, they are considered parsimony not-informative characters, although they may be useful for the distinguishing of populations [29]. In *Anopheles* mosquitoes, there are species as *An. gambiae* in which the ITS-2 shows no important length differences due to only one indel linked to species subtypes [81], whereas in others such as those of the *An. barbirostris* group a wide ITS-2 length variability was found as a consequence of different numbers of long tandem repeats [82]. In *Ae. albopictus*, the analysis of polymorphisms in ITS-2 confirms the existence of variable sites due to mutations (p-info + singleton sites), but also numerous indels, which generate consecutive regions with deletions underlying large differences in ITS-2 length both within the same individual and in different individuals of the same population. Interestingly, in the Barcelona population, considered the first *Ae. albopictus* population to arrive in Spain, none of its specimens presented the long regions of indels. In the remaining populations from Spain, France and Brazil we found specimens showing these anomalies in the length of this spacer.

To assure that a phenomenon of a pseudogenic sequence escaping from rDNA concerted evolution [40] was not involved, such a possibility was cheeked and excluded, as the 5.8S-ITS-2 sequences we amplified do not fit the characteristics of a typical pseudogene or paralogous 5.8S-ITS-2 sequence [38, 83]. Minisatellite repeats, observed to cause pronounced rDNA ITS sequence length variation in given insect species [84], were also checked for and verified to not be involved in *Ae. albopictus*. In the literature there is only one case (haplotype Aa49—[39] from St. Denis, Reunion) presenting a similar ITS-2 of reduced length and a large region of consecutive deletions appearing in the alignment with sequences from other countries.

Compared analysis with other countries allowed us to confirm that no haplotypes corresponding to the subgroup of *Ae. albopictus* cryptic species were present in our samples. When haplotypes/isolates from other eight countries were included in the analysis, the data set furnished 185 ITS-2 haplotypes and allowed us to detect:

- a reduced number of haplotypes shared between south-western Europe and Brazilian populations and other countries (8/185 = 4.3%);
- Italy shared at least 6 haplotypes with Valencia, France, Goiania and Manaus;
- two of the 3 haplotypes shared between Europe and Brazil, were also detected in Italy, USA and Thailand, illustrating genetic relationships and spread of *Ae. albopictus* around the world and mainly in areas where recent outbreaks of arboviruses and other pathogens have taken place.

Intra-individual variation in the sequence of the rDNA operon is unusual in animal genomes, although it has been reported in fishes and corals in relation to polyploidy, hybridization and a reduced rate of concerted evolution, and also in other insects [85]. In mosquitoes, intragenomic heterogeneity of rDNA spacers has been observed in *Aedes* and *Culex* [86] and in malaria *Anopheles* vectors [87, 88]. The intra-individual variation found in ITS-2 sequences of *Ae. albopictus* indicate multiple introduction events, evidenced by genetic differences with the founder population in Spain. Indeed, the Mallorca island population exhibits the highest *cox*1 haplotype diversity (Hd) and nucleotide diversity (*π*), typical of an origin population (although introduced from Asia). From the founder population, the offspring expanded along the Mediterranean coast and the Balearic Islands. In these islands, they were affected by the introduction of other lineages due to recent anthropogenic activities, as e.g. tourism and commercial flow.

The large ITS-2 haplotype variability in populations and inside specimens may be interpreted as the consequence of successive introductory events. Moreover, the result furnished by the TCS network illustrating the absence of defined haplotype groups according to geography and the interconnections between all haplotypes (Fig. 4) indicate the fast passive spreading capacity of this mosquito by means of human activities. Indeed, similarly as in the case of *cox*1, the overall negative values of Tajima’s D and *F*_*S*_ in the neutrality test indicate an excess of low frequency polymorphisms and a recent population expansion.

### Wing phenotype

Morphometric studies have allowed to infer that females of *Ae. albopictus* have a larger centroid wing size than males, in agreement with other studies in *Ae. aegypti* [89, 90]. Furthermore, a statistical difference in size variation of male *Ae. albopictus* was detected. The mosquitoes from insular Spain were significantly different from those of continental Spain/France and Brazil (*P* < 0.05). Although environmental factors, such as food quality and quantity, temperature and suitable habitat, have been evoked to underlie similar results in *Mansonia* species [91], the possible influence of the insularity phenomenon should not be overlooked [92, 93]. Molecular differences between the Spanish insular population and European or Brazilian mainland populations were evident at *cox*1 (high haplotype diversity -Hd-, and nucleotide diversity—* π*; shared haplotypes with Italy, China, Japan) and ITS-2 (high number of shared haplotypes by Brazilian and European populations, presence of long regions of deletions) levels, and are in agreement with the phenotypic results.

Regarding wing shape variation, the factor map of the landmark-based discriminant analysis derived from final shape variables allowed for the detection of two zones: one corresponding to specimens from Brazil and another to specimens from Spain (continental and insular areas) and France.

The factor map of the outline-based discriminant analysis derived from shape variables detected four separated areas corresponding to specimens from Brazil, France, continental and insular Spain, respectively, in the wing shape morpho space of male and female *Ae. albopictus*. Normally, living organisms, including mosquitoes, are adapted to their environment. The present analysis suggest that the geographical spread affected *Ae. albopictus* morphology. These results are consistent with those obtained in *Ae. aegypti* in Thailand [89]. Interestingly, the present study shows that, in *Ae. albopictus*, distances were generally longer between males than between females, which is in agreement with other analyses on wing morphometrics of three *Aedes* vectors in Thailand [59].

## Conclusions

Both rDNA and mtDNA marker sequencing has furnished very interesting results of high applied interest:

- the rDNA ITS-2 proves to be a useful marker for studies on the spread of *Ae. albopictus*, providing pronouncedly more information than the conserved mtDNA *cox*1 usually used for this kind of studies on this mosquito species;
- the rDNA ITS-2 sequence variation allows for studies at intra-individual, intra-population and inter-population levels, thus furnishing a complete overview of the evolutionary exchanges followed by a mosquito species of such a high colonization power;
- the mosquito species *Ae. albopictus* also shows nucleotide differences between the clones of the highly conserved very short 155-bp-long rDNA gene 5.8S inside an individual, additional to intra-population and inter-population variability, according to an evolutionary rate lower that in the ITS-2 (variable positions: 17 representing a 21.2%, and 136 representing a 33.1%, respectively);
- the wing morphometry proves to be a useful phenotyping marker, allowing to distinguish different populations at the level of both male and female specimens.

The pronounced ITS-2 variability found in intra-individual clones suggests hybridizations with specimens of different origin and that these hybridizations were very recent. The presence of different haplotypes inside the same individual indicates haplotype accumulation at a very high speed, as for the rDNA concerted evolution to have not yet the time to operate towards uniformization.

The large genetic diversity detected by the ITS-2 in the Spanish populations studied is surprising if it is considered that the spread of this mosquito species along the Mediterranean coast by truck transport [18] is believed to have been made by the offspring of the original, very recent first introduction in Barcelona province just in 2004 [17]. However, the ITS-2 variability found in the island of Mallorca, where it was first detected in 2012 [19], indicates additional accumulative introductions from abroad. The genetic intermixture observed in the European populations suggests multiple introductions. Such a genetic intermixture linked to a high invasion capacity has been already reported in this species, allowing to explain how it maintains genetic diversity and avoids bottleneck effects [32, 39].

The detection, for the first time, of a degree of kinship indicated by the similar genetic structure of populations of *Ae. albopictus* from Europe and Brazil should be highlighted. The existence of ITS-2 haplotypes shared by Brazil, Spain and France leaves the doors open for future tests of susceptibility and vector competence regarding strains of ZIKV and other viruses and pathogens. All in all, the results of this study indicate the need for periodic surveillance monitorings to verify that no *Ae. albopictus* with high virus transmission capacity has been introduced into Europe.

## Supplementary Information

**Additional file 1: Table S1.** Distribution of the *cox*1 sequences analysed of *Ae. albopictus* and the corresponding 27 haplotypes they provided, according to their geographical origin.

**Additional file 2: Table S2**. Pairwise distances between *cox*1 nucleotide sequences of *Ae. albopictus* populations analysed according to PAUP.

**Additional file 3: Table S3.** Phenotypic diversity of *Ae. albopictus* from south-western Europe and Brazil. Landmark-based (LB) and outline based (OB) Mahalanobis distances (LB/OB) obtained with discriminant analysis between wing shapes of females and males.

## Data Availability

The data that support the findings of this study are openly available in the GenBank database at https://www.ncbi.nlm.gov/genbank, under accession numbers MW279068–MW279079 and MW281865–MW282026.

## Abbreviations

rDNA: Nuclear ribosomal DNA
mtDNA: Mitochondrial DNA
ITSs: Internal transcribed spacers
ITS-2: Second internal transcribed spacer
*cox*1: Cytochrome *c* oxidase subunit 1 gene
*H*: Haplotype
Hd: Haplotype diversity
*π*: Nucleotide diversity
CS: Centroid size
EFA: Elliptic Fourier Analysis
ZIKV: Zika virus
