# Supplementary material for: Aedes albopictus diversity and relationships in south-western Europe and Brazil by rDNA/mtDNA and phenotypic analyses: ITS-2, a useful marker for spread studies
Source: Parasit Vectors. 2021 Jun 26;14:333. doi: 10.1186/s13071-021-04829-9 (PMC8235640; doi:10.1186/s13071-021-04829-9)
Supplement: Supplementary file 1 — Additional file 1: Table S1. Distribution of the cox1 sequences analysed of Ae. albopictus and the corresponding 27 haplotypes they provided, according to their geographical origin. [file 13071_2021_4829_MOESM1_ESM.doc]

**Table S1** Distribution of the *cox*1 sequences analysed of *Ae. albopictus* and the corresponding 27 haplotypes they provided, according to their geographical origin

| **Haplotype**  **(nº samples)** | | **Haplotype code** | **GenBank**  **Acc.No.** | **Locality**  **(nº samples)** | **Country** |
| --- | --- | --- | --- | --- | --- |
| H1 (48) | Cox1-H1  Cox1-H1  Cox1-H1  Cox1-H1  Is 14#Rim4 Hg A1a2a1  Is 19#Pav4 Hg A1a2a  Is 18#Ath2 Hg A1a2a  Is 15#Tir1 Hg A1a2a1  Is 16#Tir2 Hg A1a2a1  H3  PoMo2599  PoMo2708  PoMo2711 | | MW279068  MW279068  MW279068  MW279068  KX383929  KX383933  KX383932  KX383930  KX383931  KC690898  MN513352  MN513359  MN513361 | Valencia (20)  Barcelona (10)  Mallorca (2)  Perpignan (7)  Rimini (1)  Pavia (1)  Athens (1)  Tirana (1)  Tirana (1)  Guangzhou^a^  Oporto (1)  Algarve (1)  Algarve (1) | Spain  Spain  Spain  France  Italy  Italy  Greece  Albany  Albany  China (= Japan, Italy, USA)  Portugal  Portugal  Portugal |
| H2 (7) | Cox1-H2 | | MW279069 | Valencia (1) | Spain |
|  | Cox1-H2 | | MW279069 | Barcelona (2) | Spain |
|  | Cox1-H2 | | MW279069 | Mallorca (4) | Spain |
| H3 (15) | Cox1-H3 | | MW279070 | Barcelona (1) | Spain |
|  | Cox1-H3 | | MW279070 | Mallorca (12) | Spain |
|  | Is 8#Ces2 Hg A1a1 | | KX383923 | Cesana (1) | Italy |
|  | H17 | | KC690912 | Xinzhu, Hsinchu^a^ | Taiwan (= Italy, USA) |
| H4 (1) | Cox1-H4 | | MW279071 | Valencia (1) | Spain |
| H5 (3) | Cox1-H5 | | MW279072 | Valencia (3) | Spain |
| H6 (1) | Cox1-H6 | | MW279073 | Barcelona (1) | Spain |
| H7 (6) | Cox1-H7  Is 1#Rim1 Hg A1a1a1  Is 6#Cas1 Hg A1a1a1a  Is 7#Pav3 Hg A1a1a1a  H37  H2 | | MW279074  KX383916  KX383921  KX383922  KC690932  JQ004525 | Valencia (1)  Rimini (1)  Cassino (1)  Pavia (1)  Monmouth County, NJ^a^  New Jersey^a^ | Spain  Italy  Italy  Italy  USA (= Italy)  USA |
| H8 (5) | Cox1-H8 | | MW279075 | Mallorca (5) | Spain |
| H9 (2) | Cox1-H9  H25 | | MW279076  KC690920 | Mallorca (1)  Nagasaki^a^ | Spain  Japan |
| H10 (1) | Cox1-H10 | | MW279077 | Mallorca (1) | Spain |
| H11 (2) | Cox1-H11 | | MW279078 | Perpignan (2) | France |
| H12 (37) | Cox1-H12  Cox1-H12  Cox1-H12  Is 9#Bra  H45 | | MW279079  MW279079  MW279079  KX383924  KC690940 | Goiania (11)  Jurujuba (11)  Manaus (14)  -  Los Angeles^a^ | Brazil  Brazil  Brazil  Brazil  USA |
| H13 (5) | Is 3#Rc1 HgA1a1a1a1  Is 5#Ces1 HgA1a1a1a1  Is 2#Vir1 Hg A1a1a1a1  Is 4#Vir2 Hg A1a1a1a1  H39 | | KX383918  KX383920  KX383917  KX383919  KC690934 | Calabria (1)  Cesena (1)  Virginia (1)  Virginia (1)  Arco, Trentino^a^ | Italy  Italy  USA  USA  Italy (= USA) |
| H14 (1) | Is 10#Lam2 Hg A1b1a | | KX383925 | Hang Chat (1) | Thailand |
| H15 (1) | Is 11#Ban7 Hg A1b1a | | KX383926 | Ban Rai (1) | Thailand |
| H16 (2) | Is 12#Ath1 Hg A1b1a  H56 | | KX383927  KC690951 | Athens (1)  Texas^a^ | Greece  USA |
| H17 (2) | Is 13#Chu3  H46 | | KX383928  KC690941 | Chumphon (1)  Los Angeles^a^ | Thailand USA |
| H18 (1) | Is 20#Fo2 Hg A1a2 | | KX383934 | Foshan, China Lab strain (1) | China |
| H19 (1) | Is 21#Los1 Hg A2a | | KX383935 | Los Baños (1) | Philippines |
| H20 (3) | Is 22#Los2 Hg A2a  Is 23_Los3 Hg A2a  is 24_Los5 Hg A2a | | KX809761  KX809762  KX809764 | Los Baños (3) | Philippines |
| H21 (1) | Is 25_Los4 Hg A2a | | KX809763 | Los Baños (1) | Philippines |
| H22 (1) | Is 26_J-Wa1 Hg A1a1a | | KX809765 | Wakayama | Japan |
| H23 (1) | H2 | | KC690897 | Guangzhou^a^ | China |
| H24 (1) | H6 | | KC690901 | Guangzhou^a^ | China |
| H25 (1) | H48 | | KC690943 | Los Angeles^a^ | USA |
| H26 (1) | H62 | | KC690957 | Hawai^a^ | USA |
| H27(2) | PoMoF506  PoMoF607 | | MN513365  MN513366 | Algarve (2) | Portugal |

H, haplotype; Hg, Haplogroup; Is, Isolate; ^a^, one specimen selected by haplotype
