# Supplementary material for: Aedes albopictus diversity and relationships in south-western Europe and Brazil by rDNA/mtDNA and phenotypic analyses: ITS-2, a useful marker for spread studies
Source: Parasit Vectors. 2021 Jun 26;14:333. doi: 10.1186/s13071-021-04829-9 (PMC8235640; doi:10.1186/s13071-021-04829-9)
Supplement: Supplementary file 2 — Additional file 2: Table S2. Pairwise distances between cox1 nucleotide sequences of Ae. albopictus populations analysed according to PAUP. [file 13071_2021_4829_MOESM2_ESM.docx]

**Table S2** Pairwise distances between *cox*1 nucleotide sequences of *Ae. albopictus* populations analysed according to PAUP

_________________________________________________________________________________________________________________________________________________________________________________________________________________________

1 2 3 4 5 6 7 8 9 10 11 12 13 14 15 16 17 18 19 20 21 22 23 24 25 26 27

_________________________________________________________________________________________________________________________________________________________________________________________________________________________

1 H01 - 0.00140 0.00070 0.00070 0.00070 0.00140 0.00140 0.00140 0.00070 0.00070 0.00349 0.00140 0.00209 0.00349 0.00279 0.00349 0.00209 0.00070 0.00628 0.00558 0.00628 0.00279 0.00070 0.00070 0.004880.00140 0.00140

2 H02 2 - 0.00070 0.00209 0.00209 0.00140 0.00140 0.00279 0.00209 0.00209 0.00349 0.00140 0.00209 0.00209 0.00140 0.00209 0.00209 0.00070 0.00488 0.00419 0.00488 0.00279 0.00209 0.00140 0.003490.00279 0.00140

3 H03 1 1 - 0.00140 0.00140 0.00209 0.00070 0.00209 0.00140 0.00140 0.00279 0.00070 0.00140 0.00279 0.00209 0.00279 0.00140 0.00140 0.00558 0.00488 0.00558 0.00209 0.00140 0.00070 0.004190.00209 0.00070

4 H04 1 3 2 - 0.00140 0.00209 0.00209 0.00209 0.00140 0.00140 0.00419 0.00209 0.00279 0.00419 0.00349 0.00419 0.00279 0.00140 0.00698 0.00628 0.00698 0.00349 0.00140 0.00140 0.005580.00209 0.00209

5 H05 1 3 2 2 - 0.00209 0.00209 0.00209 0.00140 0.00140 0.00419 0.00209 0.00279 0.00419 0.00349 0.00419 0.00279 0.00140 0.00698 0.00628 0.00698 0.00349 0.00140 0.00140 0.005580.00209 0.00209

6 H06 2 2 3 3 3 - 0.00279 0.00279 0.00209 0.00209 0.00488 0.00279 0.00349 0.00349 0.00279 0.00349 0.00349 0.00070 0.00628 0.00558 0.00628 0.00419 0.00209 0.00209 0.004880.00279 0.00279

7 H07 2 2 1 3 3 4 - 0.00279 0.00209 0.00209 0.00209 0.00140 0.00070 0.00349 0.00279 0.00349 0.00209 0.00209 0.00628 0.00558 0.00628 0.00140 0.00209 0.00140 0.004880.00279 0.00140

8 H08 2 4 3 3 3 4 4 - 0.00070 0.00209 0.00488 0.00279 0.00349 0.00488 0.00419 0.00488 0.00349 0.00209 0.00628 0.00558 0.00628 0.00419 0.00209 0.00209 0.006280.00279 0.00279

9 H09 1 3 2 2 2 3 3 1 - 0.00140 0.00419 0.00209 0.00279 0.00419 0.00349 0.00419 0.00279 0.00140 0.00698 0.00628 0.00698 0.00349 0.00140 0.00140 0.005580.00209 0.00209

10 H10 1 3 2 2 2 3 3 3 2 - 0.00419 0.00209 0.00279 0.00419 0.00349 0.00419 0.00279 0.00140 0.00698 0.00628 0.00698 0.00349 0.00140 0.00140 0.005580.00209 0.00209

11 H11 5 5 4 6 6 7 3 7 6 6 - 0.00349 0.00279 0.00558 0.00488 0.00558 0.00419 0.00419 0.00837 0.00768 0.00837 0.00070 0.00419 0.00349 0.006980.00488 0.00349

12 H13 2 2 1 3 3 4 2 4 3 3 5 - 0.00209 0.00209 0.00140 0.00209 0.00070 0.00209 0.00628 0.00558 0.00628 0.00279 0.00209 0.00140 0.003490.00279 0.00140

13 H12 3 3 2 4 4 5 1 5 4 4 4 3 - 0.00419 0.00349 0.00419 0.00279 0.00279 0.00698 0.00628 0.00698 0.00209 0.00279 0.00209 0.005580.00349 0.00209

14 H14 5 3 4 6 6 5 5 7 6 6 8 3 6 - 0.00070 0.00140 0.00140 0.00279 0.00698 0.00628 0.00698 0.00488 0.00279 0.00349 0.002790.00488 0.00349

15 H15 4 2 3 5 5 4 4 6 5 5 7 2 5 1 - 0.00070 0.00070 0.00209 0.00628 0.00558 0.00628 0.00419 0.00209 0.00279 0.002090.00419 0.00279

16 H16 5 3 4 6 6 5 5 7 6 6 8 3 6 2 1 - 0.00140 0.00279 0.00698 0.00628 0.00698 0.00488 0.00279 0.00349 0.001400.00488 0.00349

17 H17 3 3 2 4 4 5 3 5 4 4 6 1 4 2 1 2 - 0.00279 0.00698 0.00628 0.00698 0.00349 0.00140 0.00209 0.002790.00349 0.00209

18 H18 1 1 2 2 2 1 3 3 2 2 6 3 4 4 3 4 4 - 0.00558 0.00488 0.00558 0.00349 0.00140 0.00140 0.004190.00209 0.00209

19 H19 9 7 8 10 10 9 9 9 10 10 12 9 10 10 9 10 10 8 - 0.00070 0.00279 0.00768 0.00698 0.00628 0.008370.00768 0.00628

20 H20 8 6 7 9 9 8 8 8 9 9 11 8 9 9 8 9 9 7 1 - 0.00209 0.00698 0.00628 0.00558 0.007680.00698 0.00558

21 H21 9 7 8 10 10 9 9 9 10 10 12 9 10 10 9 10 10 8 4 3 - 0.00768 0.00698 0.00628 0.008370.00768 0.00628

22 H22 4 4 3 5 5 6 2 6 5 5 1 4 3 7 6 7 5 5 11 10 11 - 0.00349 0.00279 0.006280.00419 0.00279

23 H23 1 3 2 2 2 3 3 3 2 2 6 3 4 4 3 4 2 2 10 9 10 5 - 0.00140 0.004190.00209 0.00209

24 H24 1 2 1 2 2 3 2 3 2 2 5 2 3 5 4 5 3 2 9 8 9 4 2 - 0.004880.00209 0.00140

25 H25 7 5 6 8 8 7 7 9 8 8 10 5 8 4 3 2 4 6 12 11 12 9 6 7 -0.00628 0.00488

26 H26 2 4 3 3 3 4 4 4 3 3 7 4 5 7 6 7 5 3 11 10 11 6 3 3 9 - 0.00279

27 H27 2 2 1 3 3 4 2 4 3 3 5 2 3 5 4 5 3 3 9 8 9 4 3 2 7 4 -

_________________________________________________________________________________________________________________________________________________________________________________________________________________________

Below diagonal, total character differences; above diagonal,mean character differences.

Haplotypes H1 to H12 correspond to *Ae. albopictus* from present paper. H13 to H27 correspond to haplotypes retrieved from GenBank. Haplotype codes and countries listed in Table S1
