# Supplementary material for: Aedes albopictus diversity and relationships in south-western Europe and Brazil by rDNA/mtDNA and phenotypic analyses: ITS-2, a useful marker for spread studies
Source: Parasit Vectors. 2021 Jun 26;14:333. doi: 10.1186/s13071-021-04829-9 (PMC8235640; doi:10.1186/s13071-021-04829-9)
Supplement: Supplementary file 3 — Additional file 3: Table S3. Phenotypic diversity of Ae. albopictus from south-western Europe and Brazil. Landmark-based (LB) and outline based (OB) Mahalanobis distances (LB/OB) obtained with discriminant analysis between wing shapes of females and males. [file 13071_2021_4829_MOESM3_ESM.doc]

**Table S3.** Phenotypic diversity of *Ae. albopictus* from south-western Europe and Brazil. Landmark-based (LB) and outline based (OB) Mahalanobis distances (LB/OB) obtained with discriminant analysis between wing shapes of females and males

|  | **Sex** | **Goiania LB/OB** | **Jurujuba LB/OB** | **Manaus LB/OB** | **Valencia LB/OB** | **Barcelona LB/OB** | **Mallorca LB/OB** |
| --- | --- | --- | --- | --- | --- | --- | --- |
| **Jurujuba** | Female | 22.41^a^/6.15 |  |  |  |  |  |
|  | Male | 4.54/2.59 |  |  |  |  |  |
| **Manaus** | Female | 13.72/4.28 | 30.18/4.00 |  |  |  |  |
|  | Male | 5.42/3.19 | 3.99/2.80 |  |  |  |  |
| **Valencia** | Female | 8.15/4.70 | 23.82/2.64 | 8.92/2.50 |  |  |  |
|  | Male | 6.40/3.23 | 5.28/2.51 | 4.57/2.25 |  |  |  |
| **Barcelona** | Female | 9.45/3.41 | 26.72/5.40 | 10.37/3.28 | 7.62/3.97 |  |  |
|  | Male | 6.43/2.48 | 4.70/1.86 | 6.13/2.67 | 5.27/2.37 |  |  |
| **Mallorca** | Female | 14.50/4.29 | 33.53/4.92 | 7.97/2.41 | 11.31/3.61 | 9.58/2.72 |  |
|  | Male | 6.55/3.40 | 4.34/2.63 | 5.85/2.98 | 5.07/1.37 | 5.54/2.26 |  |
| **Perpignan** | Female | 10.39/4.04 | 28.75/4.80 | 7.92/3.13 | 7.32/3.72 | 5.77/2.32 | 6.31/1.87 |
|  | Male | 7.73/4.69 | 6.62/3.70 | 7.49/3.42 | 7.34/2.67 | 6.76/3.74 | 6.06/3.11 |

^a^ statistically significant pairwise Mahalanobis distances
